# Two New Phomaligols from the Marine-Derived Fungus *Aspergillus flocculosus* and Their Anti-Neuroinflammatory Activity in BV-2 Microglial Cells

**DOI:** 10.3390/md19020065

**Published:** 2021-01-27

**Authors:** Byeoung-Kyu Choi, Duk-Yeon Cho, Dong-Kug Choi, Phan Thi Hoai Trinh, Hee Jae Shin

**Affiliations:** 1 Marine Natural Products Chemistry Laboratory, Korea Institute of Ocean Science and Technology, 385 Haeyang-ro, Yeongdo-gu, Busan 49111, Korea; choibk4404@kiost.ac; 2 Department of Applied Life Science, Graduate School, BK21 Program, Konkuk University, Chungju 27478, Korea; whejrdus10@kku.ac.kr (D.-Y.C.); choidk@kku.ac.kr (D.-K.C.); 3 Nhatrang Institute of Technology Research and Application, Vietnam Academy of Science and Technology, 02 Hung Vuong, Nha Trang 650000, Vietnam; phanhoaitrinh@nitra.vast.vn

**Keywords:** phomaligol, marine fungus, *Aspergillus flocculosus*, BV-2 microglia, anti-neuroinflammatory

## Abstract

Two new phomaligols, deketo-phomaligol A (**1**) and phomaligol E (**2**), together with six known compounds (**3**–**8**) were isolated from the culture broth of the marine-derived fungus *Aspergillus flocculosus*. Compound **1** was first isolated as a phomaligol derivative possessing a five-membered ring. The structures and absolute configurations of the new phomaligols were determined by detailed analyses of mass spectrometry (MS), nuclear magnetic resonance (NMR) data, optical rotation values and electronic circular dichroism (ECD). In addition, the absolute configurations of the known compounds **3** and **4** were confirmed by chemical oxidation and comparison of optical rotation values. Isolated compounds at a concentration of 100 μM were screened for inhibition of nitric oxide (NO) production in lipopolysaccharide (LPS)-induced BV-2 microglial cells. Among the compounds, **4** showed moderate anti-neuroinflammatory effects with an IC_50_ value of 56.6 μM by suppressing the production of pro-inflammatory mediators in activated microglial cells without cytotoxicity.

## 1. Introduction

Marine environment is considered as a rich source of novel compounds having chemically diverse and complex structures, as it is not only extremely broad and untapped area, but also diverse and unique habitats such as salinity, temperature, and extreme pressure [1,2]. Marine microorganisms have evolved the ability to produce secondary metabolites to adapt to various environments, to protect themselves from predators, to communicate (quorum sensing) each other, and so on [3,4]. Over the past decade, various chemical sources from marine microbes have been researched for drug discovery and development [5]. Among the marine organisms, marine-derived fungi produce bioactive compounds that can be considered to display a wide range of bioactivities including antimicrobial, anti-inflammatory, antiplasmodial and anticancer [6,7]. 

**Figure 1:**
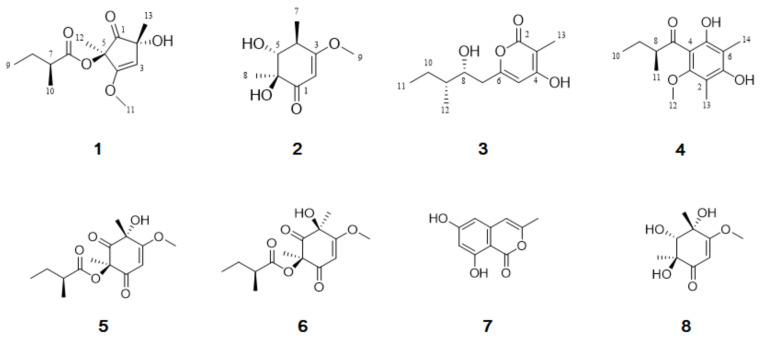
Structures of **1**–**8** isolated from *Aspergillus flocculosus*.

Neurodegenerative diseases such as Alzheimer’s disease and Parkinson’s disease are associated with chronic neuroinflammation caused by a high production of several inflammatory factors including nitric oxide (NO), reactive oxygen species (ROS), tumor necrosis factor alpha (TNF-α), interleukin-1 beta (IL-1β) and interleukin-6 (IL-6) [8,9,10]. Microglia are the resident macrophages of the brain to respond to brain injury or infection [11]. Pathological microglial activation triggers the inflammatory response, which is believed to be involved in neuroinflammatory processes [12]. Therefore, the suppression of pro-inflammatory mediators in activated microglia may lead the development of therapeutic agents for various neuronal diseases [13]. During our ongoing investigation for new metabolites and biological activities from marine microorganisms, we encountered a fungal strain 168ST-16.1 which produces diverse secondary metabolites. The fungal strain was fermented and extracted with EtOAc, and then the extract was evaporated under reduced pressure to yield a crude extract, which was fractionated by flash column chromatography on ODS using mixtures of MeOH/H_2_O. The fractions were purified by reversed-phase HPLC to afford two new compounds (**1** and **2**) together with six known compounds, sydowione A (**3**) [14], 2,6-dimethyl-3-*O*-methyl-4-(2-methylbutyryl) phloroglucinol (**4**) [15], phomaligol A (**5**) [16], phomaligol A_1_ (**6**) [16], saccharonol A (**7**) [17], and phomaligol D (**8**) [18] (Figure 1). Their structures were elucidated by spectroscopic methods (1D, 2D NMR and HRESIMS), modified Mosher’s method, acid hydrolysis and comparison of specific rotation values with the literature data as shown in Figure 1. All the isolated compounds were evaluated for their effects on nitric oxide production in lipopolysaccharide (LPS)-stimulated murine microglia BV-2 cells. Herein, the isolation and structural elucidation of compounds **1**–**8** and their biological activities are described.

## 2. Results and Discussion

**Figure 2:**
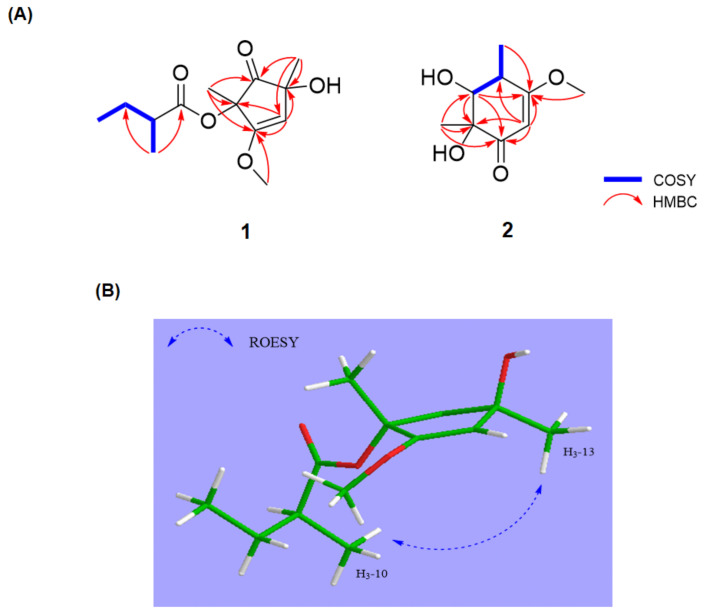
(**A**) Key COSY and HMBC correlations of **1** and **2**. (**B**) Key ROESY correlations of **1**.

Compound **1** was isolated as a pale yellow oil and its molecular formula was determined to be C_13_H_20_O_5_ based on HRESIMS (279.1209, [M + Na]^+^). The ^1^H NMR spectrum of **1** showed the signals of a singlet olefinic proton at δ_H_ 4.99 (s, 1H), a methine at δ_H_ 2.37 (m, 1H), a methylene at δ_H_ 1.46, 1.63 (m, 2H), and five methyl protons at δ_H_ 4.16 (s, 3H), 1.66 (s, 3H), 1.29 (s, 3H), 1.11 (d, J = 7.0 Hz, 3H), and 0.93 (t, J = 7.0 Hz, 3H) (Table 1). The combination of ^13^C NMR and HSQC spectra exhibited 13 carbon signals, indicating the presence of one ketone at δ_C_ 200.2 (C-1), one carbonyl at δ_C_ 175.9 (C-6), three quaternary carbons at δ_C_ 178.4 (C-4), 114.1 (C-5), and 84.9 (C-2), one sp^2^ carbon at δ_C_ 72.1 (C-3), one methine, methylene and five methyl carbons at δ_C_ 40.7 (C-7), δ_C_ 26.4 (C-8), δ_C_ 10.4 (C-9), δ_C_ 15.4 (C-10), δ_C_ 57.7 (C-11), δ_C_ 4.6 (C-12), and δ_C_ 18.1 (C-13), respectively (Table 1). The planar structure of **1** was elucidated by analysis of the 2D NMR data, including the COSY and HMBC spectra (Figure 2A). The HMBC correlations from δ_H_ 1.29 (H-13) to C-1, C-2, and C-3, δ_H_ 1.66 (H-12) to C-1, C-4, and C-5, δ_H_ 4.99 (H-3) to C-2, C-4, and C-5 and δ_H_ 4.16 (H-11) to C-4 confirmed the presence of a five-membered ring system with a ketone group. A *sec*-butyl moiety connecting to the carbonyl unit at C-7 was determined by the COSY correlations of H-7/H_2_-8/H_3_-9/H_3_-10 and the HMBC correlations from H_3_-10 and H_2_-8 to C-10. Although there was no HMBC correlation between the five-membered ring and the side chain, the ROESY correlation of H_3_-10/H_3_-13 and the molecular formula determined by HRESIMS analysis suggested that two partial structures are connected by an ester linkage. The planar structure of **1** was closely similar to phomaligols A and A_1_, except for the absence of one ketone and having a five-membered ring system. 

The stereochemistry of **1** was determined by the analysis of ROESY spectra, electronic circular dichroism (ECD) and acid hydrolysis. To elucidate the absolute configuration of C-7, compound **1** was subjected to a chemical degradation. The acid hydrolysis of **1** afforded 2-methylbutanoic acid, and a specific rotation value of the hydrolysate was compared with reference compounds, (+) and (−)-2-methylbutanoic acids (Supplementary Materials Figure S8). The positive optical rotation value (${\left[\alpha \right]}_{D}^{25}$ +20.0 (*c* 0.1, MeOH) of 2-methylbutanoic acid in **1** suggested the absolute configuration of C-7 is (*S*), same as the isolated phomaligols **5** and **6**.

**Figure 3:**
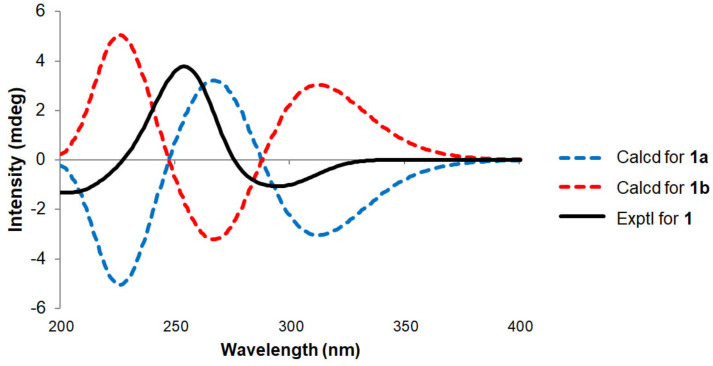
Comparison of the circular dichroism (CD) curves between the experimental and calculated data of **1**.

Afterward, the relative configurations of C-2 and C-5 were established by ROESY correlations. The strong correlation signals of H_3_-10/H_3_-13 suggested that the *sec*-butyl moiety and H_3_-13 were on the same face, which was also supported by the lack of ROESY correlation of H_3_-12/H_3_-13 (Figure 2B). Based on the above correlations, there are only two possible conformers (**1a**: 2*R*, 5*R*, 7*S*, **1b**: 2*S*, 5*S*, 7*S*). Finally, to determine the absolute stereochemistry of **1**, ECD calculation of the possible conformers was carried out at B3LYP/6-311+G(d,p) level. As shown in Figure 3, the calculated ECD spectra of **1a** and **1b** displayed the opposite pattern even though two possible structures are not enantiomers. The calculated ECD spectrum of **1** was in a better agreement with the experimental CD spectrum of **1a**, suggesting the absolute configuration of **1** is defined as 2*R*, 5*R*, 7*S*. To the best of our knowledge, **1** is the first phomaligol with a five-membered ring and is named deketo-phomaligol A.

Compound **2** was purified as a pale yellow oil with a molecular formula of C_9_H_14_O_4_ by HRESIMS (209.0791, [M + Na]^+^). ^1^H and ^13^C NMR data of **2** were similar to those of phomaligol D (**8**) except for the presence of a multiplet methine at δ_H_ 3.19 (H-4), a doublet methine at δ_H_ 3.74 (H-5) and a doublet methyl proton at δ_H_ 1.23 (H-7). The COSY correlations of H-4/H-5/H-7 and HMBC correlations from H-2 to C-1, C-3, C-4 and C-6, H_3_-7 to C-3 and H-5 to C-1, C-3 and C-6 suggested the presence of a cyclohexanone as shown in Figure 2A. Subsequently, the HMBC correlations from H_3_-8 to C-1, C-5 and C-6 and H_3_-9 to C-3 confirmed the structure of **2** to be a phomaligol derivative, differing only in a hydroxyl group at C-4 compared to **8**. 

The relative configurations of two hydroxyl groups and two singlet methyls were elucidated by comparison of chemical shifts with the known phomaligol and ROESY correlations. Almost identical chemical shift of H_3_-8 between **2** (δ_H_ 1.35) and **8** (δ_H_ 1.36) suggested that two hydroxyl groups near H_3_-8 in **2** could be in the same chemical environment as in **8**. In addition, the presence of H-5 and H_3_-7 on the same face was confirmed by the ROESY correlations between H-5 and H_3_-7. These results suggested the relative configurations of **2** and **8** are identical. Finally, the same absolute configuration of **2** as that of **8** was determined by the comparison of the specific rotation values of **2** (${\left[\alpha \right]}_{D}^{25}$−60.0 (*c* 0.4, MeOH)) and **8** (${\left[\alpha \right]}_{D}^{25}$−55.6 (*c* 0.4, MeOH)). Therefore, the structure of **2** was elucidated and named phomaligol E (**2**).

Planar structures of **3** and **4** were determined by detailed NMR analyses as sydowione A and 2,6-dimethyl-3-O-methyl-4-(2-methylbutyryl) phloroglucinol, respectively. However, the known compounds were published without deciphering full absolute configurations. Here, we report the elucidation of the absolute configurations of compounds **3** and **4**. 

**Figure 4:**
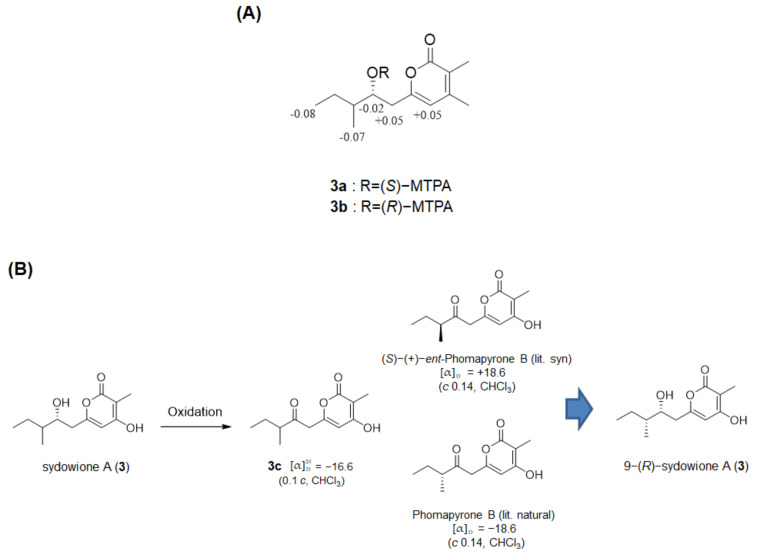
(**A**) Δ*_S_*-*_R_* values in ppm of the MTPA esters of **3**. (**B**) Assignment of absolute configuration of C-9 in **3**.

The absolute configuration of **3** was confirmed by modified Mosher’s method, oxidation and comparison of specific rotation values. To determine the absolute configuration of C-8, **3** was subjected to the modified Mosher’s method. The observed chemical shift differences Δδ*_S_*_−*R*_ was calculated to assign the 8*R* configuration in **3** (Figure 4A). Afterward, the stereochemistry of C-9 was elucidated by comparison of specific rotation values with a similar compound, phomapyrone B, which has been reported for its total synthesis and enantiomer so far [19]. The only difference between **3** and phomapyrone B is the presence of a ketone or a hydroxyl group at C-9. Consequently, chemical oxidation of secondary alcohol at C-8 to ketone in **3** led to the production of phomapyrone B (**3c**) and determination of absolute configuration at C-9 by comparing the measured optical rotation value ${\left[\alpha \right]}_{D}^{20}$−16.6 (*c* 0.1, CHCl_3_) with the literature (phomapyrone B, [α]_D_ −18.6 (*c* 0.14, CHCl_3_)(Figure 4B and Supplemetary Materials Figure S20). Thus, the absolute stereochemistry of **3** was determined as 9-(*R*)-sydowione A.

**Figure 5:**
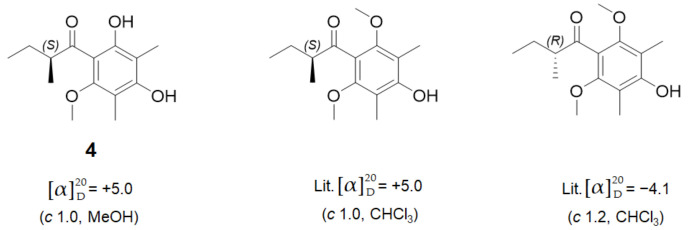
Comparison of the optical rotation value of **4** with reference compounds.

The absolute configuration of **4** was elucidated by comparison of optical rotation values with those of reported similar compounds, which have the same backbone with **4**, differing by only an additional methoxy group. Based on a literature search, the optical rotation values of the congeners represent a negative or positive value depending on the stereochemistry of the *sec*-butyl moiety (Figure 5) [20,21]. According to the optical rotation value ${\left[\alpha \right]}_{D}^{20}$+5.0 (*c* 1.0, MeOH), the absolute configuration of C-8 in **4** was confirmed as (*S*). 

**Figure 6:**
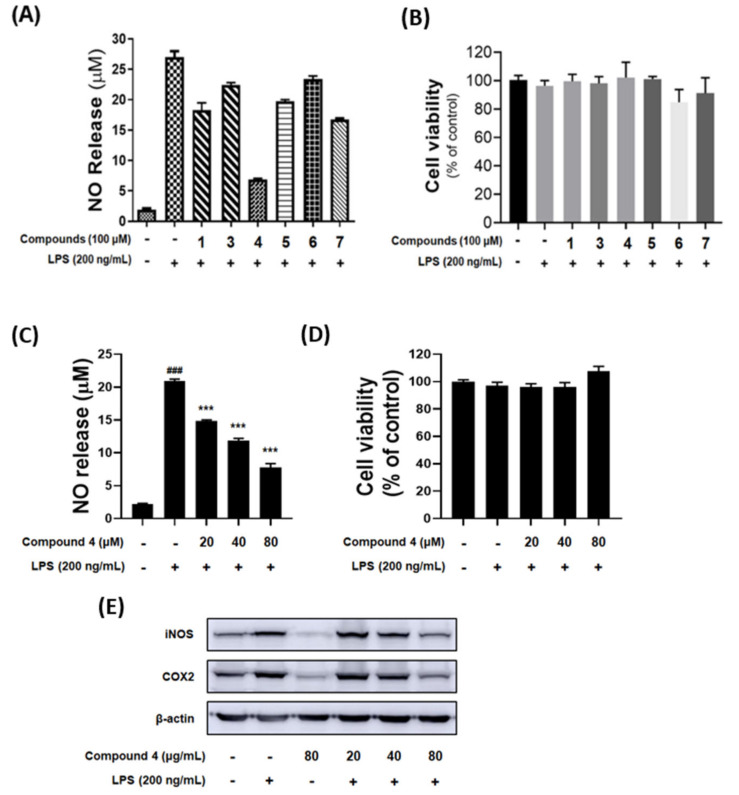
(**A**) The measurements of nitrite levels in the culture media were conducted on the Griess reaction. **(B)** Cell viability was tested using the MTT assay. **(C)** The measurements of nitrite levels and **(D)** Cell viability of **4** were tested at a concentration of 20, 40 and 80 μM. (**E**) Inhibitory effects of iNOS and COX-2 protein expression by compound **4** in LPS-stimulated BV-2 cells. Values are mean ± standard error. ^###^ *p* < 0.001, vs. control group and *** *p* < 0.001 vs. LPS-treated group.

Compounds **1** and **3**~**7** were tested for their anti-neuroinflammatory effects in LPS-induced BV-2 microglia cells and cytotoxicity. Each compound was treated with 100 μM to evaluate the levels of the LPS-induced (200 ng/mL) NO production in BV-2 microglial cells. Interestingly, compound **4** inhibited NO production in BV-2 microglial cells without cytotoxicity as shown in Figure 6A,B. To investigate the regulation of LPS-induced NO production and expression levels of iNOS and COX-2 proteins, compound **4** at the concentrations of 20, 40 and 80 μM was evaluated, and the results showed that **4** reduced the NO production and significantly downregulated the expression of iNOS and COX-2 proteins in a dose-dependent manner (Figure 6C–E). 

## 3. Materials and Methods 

### 3.1. General Experimental Procedures 

Optical rotations were acquired on a Rudolph Research Analytical Autopol III polarimeter (Rudolph Research Analytical, Hackettstown, NJ, USA). NMR spectra were collected on a Varian Unity 500 MHz (Varian Inc., Palo Alto, CA, USA) and a Bruker 600 MHz spectrometer (Bruker BioSpin GmbH, Rheinstetten, Germany). HRESIMS were recorded on Waters Synapt HDMS LC/MS mass spectrometer (Waters Corporation, Milford, MA, USA). IR spectra were measured on a JASCO FT/IR-4100 spectrophotometer (JASCO Corporation, Tokyo, Japan). CD spectra were obtained on a JASCO J-1500 spectrometer (JASCO Corporation, Tokyo Japan). HPLC was performed with PrimeLine Binary pump (Analytical Scientific Instruments, Inc., El Sobrante, CA, USA) and RI-101 (Shoko Scientific Co. Ltd., Yokohama, Japan). Column chromatography was performed using ODS gel (12 nm, S-75 μM, YMC CO., Kyoto, Japan). Semi-preparative HPLC was carried out with an ODS column (YMC-Pack-ODS-A, 250 × 10 mm i.d, 5 μM). Analytical HPLC was performed using an ODS column (YMC-Pack-ODS-A, 250 × 4.6 mm i.d, 5 μM).

### 3.2. Fungal Material and Fermentation 

The fungus *Aspergillus flocculosus* 168ST-16.1 was isolated from the algae *Padina* sp., collected using SCUBA at a depth of 10 m in Son Tra peninsular, Da Nang, Vietnam and cultured on rice media at 28 °C for three weeks in Erlenmeyer flasks, each containing rice, yeast extract, KH_2_PO_4_, and natural sea water as previously described [22].

### 3.3. Isolation of Compounds ***1***–***8***

The mycelia and medium were homogenized and extracted with EtOAc and then concentrated in vacuo to yield a crude extract (22 g). The crude extract was fractionated by flash column chromatography on C_18_-reversed phase silica gel (ODS) using a gradient of MeOH/ H_2_O (*v/v* 1:4 to 100% MeOH, each fraction 300 mL) to yield 15 fractions (Fr.A-Fr.O). The Fr. E (1.3 g) was further chromatographed into ten subfractions (Fr. E.1–10) by ODS eluting with a step gradient of MeOH/H_2_O (30:70 to 40:60, *v/v*). Fr. E2 (210 mg) was purified by a semi-preparative reversed-phase HPLC (4.0 mL/min, RI detector) using isocratic elution with 38% MeOH in H_2_O to yield **1** (3.5 mg, *t*_R_ = 34 min) and **6** (2.7 mg, *t*_R_ = 36 min). Compounds **3** (8.2 mg, *t*_R_ = 46 min) and **5** (4.1 mg, *t*_R_ = 50 min) were isolated from the Fr. F (894 mg) by a semi-preparative reversed-phase HPLC (38% MeOH/H_2_O, RI detector, 4.0 mL/min). The Fr. A (1.3 g) was purified with an analytical reversed-phase HPLC (10% MeOH/H_2_O, RI detector, 1.0 mL/min) to afford **8** (1.6 mg, *t*_R_ = 15 min). Compound **2** (1.5 mg, *t*_R_ = 25 min) was obtained from the Fr. B (350 mg) by an analytical reversed-phase HPLC (12% MeOH/H_2_O, RI detector, 1.0 mL/min). The Fr. H (3.7 g) was further purified through a semi-preparative reversed-phase HPLC (4.0 mL/min, RI detector, 50% MeOH/H_2_O) to give **7** (2.6 mg, *t*_R_ = 14 min). Compound **4** (4.3 mg, *t*_R_ = 24 min) was separated from the Fr. L (897 mg) by a semi-preparative reversed-phase HPLC (65% MeOH/H_2_O, RI detector, 4.0 mL/min). All the procedures for the fractionation and isolation of the compounds were performed according to previously reported techniques [23].

### 3.4. Spectral Data 

Deketo-phomaligol A (**1**): pale yellow oil; ${\left[\alpha \right]}_{D}^{25}$+33.0(*c* 0.05, MeOH); IR νmax 3328, 1626, 1658, 1384, 1328, 1056 cm^−1^; UV(MeOH) λ_max_ (log ε) 253 (3.54), 208 (3.22) nm; HRESIMS *m/z* 279.1209 [M + Na]^+^ (calcd for 279.1208, C_13_H_20_O_5_Na); ^1^H NMR (CD_3_OD, 500 MHz) and ^13^C NMR (CD_3_OD, 125 MHz) see Table 1.

Phomaligol E (**2**): pale yellow oil; ${\left[\alpha \right]}_{D}^{25}$−60.0(*c* 0.4, MeOH); IR νmax 3388, 1643, 1593, 1455, 1374, 1225, 1056 cm^−1^; UV(MeOH) λ_max_ (log ε) 255 (3.25), 203 (3.10) nm; HRESIMS *m/z* 209.0791 [M + Na]^+^ (calcd for 209.0790, C_9_H_14_O_4_Na); ^1^H NMR (CD_3_OD, 500 MHz) and ^13^C NMR (CD_3_OD, 125 MHz) see Table 1.

### 3.5. Calculation of ECD Spectra

Conformational searches and theoretical calculation for ECD spectra were performed by conflex version 8.0 (CONFLEX Corporation, Tokyo, Japan) and Gaussian 16 software (Gaussian Inc., Wallingford, CT, USA). Optimization of conformers and theoretical calculations of ECD data were performed using the time-dependent density functional theory (TD-DFT) method at the B3LYP/6-311G+(d,p) according to previously reported procedures [24].

### 3.6. Preparation of MTPA and Esters of ***3*** Using the Mosher’s Method

(*R*)- or (*S*)-MTPA-Cl and anhydrous pyridine were added to compound **3**, and the reaction mixture was stirred overnight at room temperature. The procedures for the absolute configuration determination using the Mosher’s method were performed as previously reported [25].

Compound **3a**: ^1^H NMR (CD_3_OD, 500 MHz) *δ*_H_ 6.34 (1H, s, H-5), *δ*_H_ 5.49 (1H, m, H-8), *δ*_H_ 2.92 (2H, d, H-7), *δ*_H_ 1.76 (3H, s, H-13), *δ*_H_ 0.93 (3H, d, H-12), *δ*_H_ 0.87 (3H, t, H-11); ESIMS *m/z* 481.3 [M + Na]^+^

Compound **3b**: ^1^H NMR (CD_3_OD, 500 MHz) *δ*_H_ 6.29 (1H, s, H-5), *δ*_H_ 5.51 (1H, m, H-8), *δ*_H_ 2.87 (2H, d, H-7), *δ*_H_ 1.76 (3H, s, H-13), *δ*_H_ 1.01 (3H, d, H-12), *δ*_H_ 0.94 (3H, t, H-11); ESIMS *m/z* 481.3 [M + Na]^+^

### 3.7. Hydrolysis of ***1***, and Oxidation of ***3*** for Determination of Absolute Configuration

Compound **1** was dissolved in 6N HCl (0.5 mL) and heated to 100 ℃ for 1 h. The solution was cooled and extracted with EtOAc twice. The EtOAc layer was concentrated under reduced pressure. The extract was chromatographed by ODS using a stepwise elution with combinations of MeOH/H_2_O (*v/v* 1:4, 2:3, 3:2, 4:1 and 100% MeOH). The MeOH/H_2_O (2:3) and MeOH/H_2_O (1:4) fractions gave a 2-methylbutanoic acid (Supporting information). 

To determine the absolute configuration, the secondary alcohol of **3** (1.5 mg) in dichloromethane (0.5 mL) was oxidized with pyridinium dichromate (3 eqiv.) at room temperature overnight. After work-up, the extract was purified by analytical HPLC (UV detector, flow rate 1.0 mL/min,) using a gradient elution from 10% to 100% MeOH in 60 min to yield **3c** (*t*_R_ = 19 min).

Compound **3c**: ^1^H NMR (CD_3_OD, 600 MHz) *δ*_H_ 5.85 (1H, s, H-5), *δ*_H_ 3.28 (2H, overlap, H-7), *δ*_H_ 2.66 (1H, m, H-9), *δ*_H_ 1.80 (3H, s, H-13), *δ*_H_ 1.69, 1.40 (2H, m, H-10), *δ*_H_ 1.07 (3H, d, H-12), *δ*_H_ 1.88 (3H, t, H-11); ESIMS *m/z* 247.2 [M + Na]^+^

### 3.8. BV-2 Microglial Cell Culture, Cell Viability, Nitrite Assay and Western Blot Analysis

Murine microglial (BV-2) cells were cultured as previously reported [24]. The BV-2 microglial cells were pretreated with isolated compounds for 1 h, followed by LPS (200 ng/mL) for 24 h. After addition of 20 μM MTT solution to 24 wells, the supernatant dissolved the formazan crystals in viable cells from DMSO was evaluated using a microplate reader at 550 nm and values were estimated in comparison to control cells. To conduct the nitrite assay, BV-2 microglial cells were pretreated with isolated compounds for 1 h, followed by LPS (200 ng/mL) for 24 h. The supernatant transferred to new microplates was mixed with Griess reagent for 10 min at room temperature in the dark. The measurement of nitrite was performed using a range of sodium nitrite dilutions as standard solutions. The absorbance was analyzed using a microplate reader at 540 nm. Western blot analysis was conducted to detect the expression of iNOS and COX-2 as described in the previous study [24]. 

## 4. Conclusions

Two new phomaligol derivatives (**1** and **2**), along with seven known compounds (**3**–**8**), were isolated from the rice medium culture of the marine-derived fungus *Aspergillus flocculosus*. To the best of our knowledge, compound **1** is the first phomaligol with a five-membered ring. Additionally, full absolute configurations of the known compounds **3** and **4** were first elucidated by acid hydrolysis, chemical oxidation and comparison of specific rotation values with reported data. Compounds **1** and **3**–**7** were tested for inhibitory activity on NO production in LPS-stimulated BV-2 microglial cells. Interestingly, **4** suppressed the production of NO and expression levels of iNOS and COX-2 proteins in a concentration-dependent manner. Consequently, these results indicated that compound **4** obtained from the marine-derived fungus *A. flocculosus* possesses effective properties against neuroinflammation in activated microglial cells without cytotoxicity.

## Figures and Tables

**Table 1:** ^1^H and ^13^C NMR data for **1, 2, 3** and **4** in CD_3_OD (500 MHz for ^1^H and 125 MHz for ^13^C).

Position	1		2		3		4	
	*δ*_H_ (*J* in Hz)	*δ*_C_	*δ*_H_ (*J* in Hz)	*δ*_C_	*δ*_H_ (*J* in Hz)	*δ*_C_	*δ*_H_ (*J* in Hz)	*δ*_C_
1		200.2		199.7				160.4
2		84.9	5.31, s	97.9		167.7		109.5
3	4.99, s	72.1		178.5		97.7		158.5
4		178.4	3.19, m	35.8		166.4		107.5
5		114.1	3.74 (d, 3.5)	76.7	6.06, s	101.4		160.5
6		175.9		73.8		161.3		107.0
7	2.37, m	40.7	1.23 (d, 7.0)	11.2	2.51 (dd, 14.5, 9.5) 2.62 (dd, 14.5, 4.0)	38.5		210.7
8	1.46, 1.63, m	26.4	1.35, s	19.6	3.89, m	71.2	3.74, m	45.0
9	0.93 (t, 7.0)	10.4	3.75, s	55.3	1.42, m	40.2	1.39, 1.76, m	27.1
10	1.11 (d, 7.0)	15.4			1.23, 1.55, m	25.5	0.87 (t, 7.5)	10.8
11	4.16, s	57.7			0.95 (t, 8.0)	10.7	1.13 (d, 6.5)	16.2
12	1.66, s	4.6			0.94 (d, 7.0)	12.4	3.67, s	61.3
13	1.29, s	18.1			1.86, s	6.8	2.09, s	7.8
14							2.02, s	6.7

The assignments were aided by ^1^H–^1^H COSY, ROESY, HSQC, and HMBC NMR spectra.

## Data Availability

The Data presented in the article are available in the supplementary materials.

## Supplementary Materials

The followings are available online at https://www.mdpi.com/1660-3397/19/2/65/s1, Figures S1–S8: the analyzed data of ^1^H and ^13^C NMR, 2D NMR (COSY, HSQC, HMBC, ROESY), HRESI-MS and chemical reaction of **1**, Figures S9–S15: ^1^H and ^13^C NMR, 2D NMR (COSY, HSQC, HMBC, ROESY) spectra and HRESI-MS data of **2**, Figures S16–S23: 1H and ^13^C NMR, HRESI-MS and chemical reaction data of **3** and **4**, Figures S24–S35: ^1^H and ^13^C NMR spectra and LRMS data of **5**–**8**, Figure S36, Tables S1–S6: ECDs of **1**.
