# Supplementary material for: Two New Phomaligols from the Marine-Derived Fungus Aspergillus flocculosus and Their Anti-Neuroinflammatory Activity in BV-2 Microglial Cells
Source: Mar Drugs. 2021 Jan 27;19(2):65. doi: 10.3390/md19020065 (PMC7911895; doi:10.3390/md19020065)
Supplement: Supplementary file 1 [file marinedrugs-19-00065-s001.pdf]

Supplementary data

Two New Phomaligols from the Marine-Derived Fungus *Aspergillus flocculosus* and their Anti-Neuroinflammatory Activity in BV-2 Microglial Cells

Byeoung-Kyu Choi[1], Duk-Yeon Cho[2], Dong-Kuk Choi[2], Phan Thi Hoai Trinh[3], and Hee Jae Shin[1,*]

1 Marine Natural Products Chemistry Laboratory, Korea Institute of Ocean Science and Technology, 385 Haeyang-ro, Yeongdo-gu, Busan 49111, Korea

2 Department of Applied Life Science, Graduate school of Konkuk University, Chungju 27478, Republic of Korea

3 Nhatrang Institute of Technology Research and Application, Vietnam Academy of Science and Technology, 02 Hung Vuong, Nha Trang, Vietnam

*Corresponding author. Tel.: +82-51-664-3341; fax: +82-51-664-3340; e-mail: shinhj@kiost.ac.kr

Contents

Figure S1. HRESIMS data of deketo-phomaligol A (**1**). ---1

Figure S2. ^{1}H NMR spectrum of deketo-phomaligol A (**1**). --2

Figure S3. ^{13}C NMR spectrum of deketo-phomaligol A (**1**). ---------------------------------------2

Figure S4. ^{1}H-^{1}H COSY spectrum of deketo-phomaligol A (**1**). ----------------------------------3

Figure S5. HSQC spectrum of deketo-phomaligol A (**1**). ---4

Figure S6. HMBC spectrum of deketo-phomaligol A (**1**). --5

Figure S7. ROESY spectrum of deketo-phomaligol A (**1**). ---6

Figure S8. ^{1}H NMR spectrum of 2-methylbutanoic acid by hydrolysis of **1.** -----------------7

Figure S9. HRESIMS data of phomaligol E (**2**). ---8

Figure S10. ^{1}H NMR spectrum of phomaligol E (**2**). --9

Figure S11. ^{13}C NMR spectrum of phomaligol E (**2**). ---9

Figure S12. ^{1}H-^{1}H COSY spectrum of phomaligol E (**2**). --10

Figure S13. HSQC spectrum of phomaligol E (**2**). --11

Figure S14. HMBC spectrum of phomaligol E (**2**). ---12

Figure S15. ROESY spectrum of phomaligol E (**2**). --13

Figure S16. HRESIMS data of sydowione A (**3**). ---14

Figure S17. ^{1}H NMR spectrum of sydowione A (**3**). ---15

Figure S18. ^{13}C NMR spectrum of sydowione A (**3**). --15

Figure S19. ^{1}H NMR spectrum of (*S*) and (*R*) MTPA (**3a** and **3b**). ---------------------------16

Figure S20. ^{1}H NMR spectrum of oxidation of **3** (**3c**). --17

Figure S21. LRMS data of 2,6-dimethyl-3-O -methyl-4-(2-methylbutyryl) phloroglucinol (**4**). --18

Figure S22. ^{1}H NMR spectrum of 2,6-dimethyl-3-O -methyl-4-(2-methylbutyryl)phloroglucinol (**4**). ---19

Figure S23. ^{13}C NMR spectrum of 2,6-dimethyl-3-O -methyl-4-(2-methylbutyryl)phloroglucinol (**4**). ---19

Figure S24. LRMS data of phomaligol A (**5**). ---20

Figure S25. ^{1}H NMR spectrum of phomaligol A (**5**). ---21

Figure S26. ^{13}C NMR spectrum of phomaligol A (**5**). --21
Figure S27. LRMS data of phomaligol A_1 (**6**). --22
Figure S28. ^{1}H NMR spectrum of phomaligol A_1 (**6**). ---23
Figure S29. ^{13}C NMR spectrum of phomaligol A_1 (**6**). --23
Figure S30. LRMS data of saccharonol A (**7**). --24
Figure S31. ^{1}H NMR spectrum of saccharonol A (**7**). --25
Figure S32. ^{13}C NMR spectrum of saccharonol A (**7**). ---25
Figure S33. LRMS data of phomaligol D (**8**). ---26
Figure S34. ^{1}H NMR spectrum of phomaligol D (**8**). ---27
Figure S35. ^{13}C NMR spectrum of phomaligol D (**8**). ---27
Figure S36. DFT optimized conformers and populations of **1** (2*R*, 5*R*, 7*S*) above 5% population. ---28
Table S1. Gibbs free energies and Boltzmann distribution of conformers of compound **1**. --28
Table S2−S6. ECD calculation and energy minimized coordinates of conformer **1**−**5** for all atoms (Å). ---29

Elemental Composition Report

Single Mass Analysis
Tolerance = 10.0 PPM / DBE: min = -1.5, max = 50.0
Element prediction: Off
Number of isotope peaks used for i-FIT = 3

Monoisotopic Mass, Even Electron Ions
39 formula(e) evaluated with 2 results within limits (up to 50 closest results for each mass)
Elements Used:
C: 0-15 H: 0-30 O: 0-10 Na: 0-1

Minimum:				-1.5				
Maximum:		5.0	10.0	50.0				
Mass	Calc. Mass	mDa	PPM	DBE	i-FIT	Norm	Conf(%)	Formula
279.1209	279.1208	0.1	0.4	3.5	1477.8	0.006	99.41	C13 H20 O5 Na

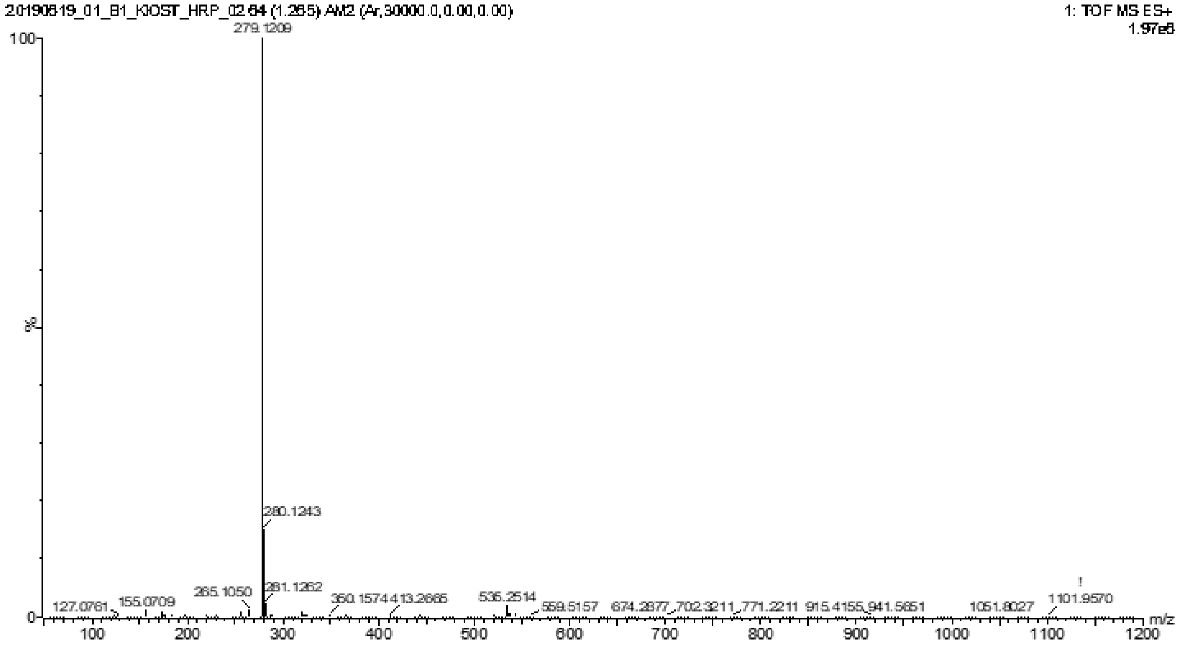

Figure S1. HRESIMS data of deketo-phomaligol A (**1**).

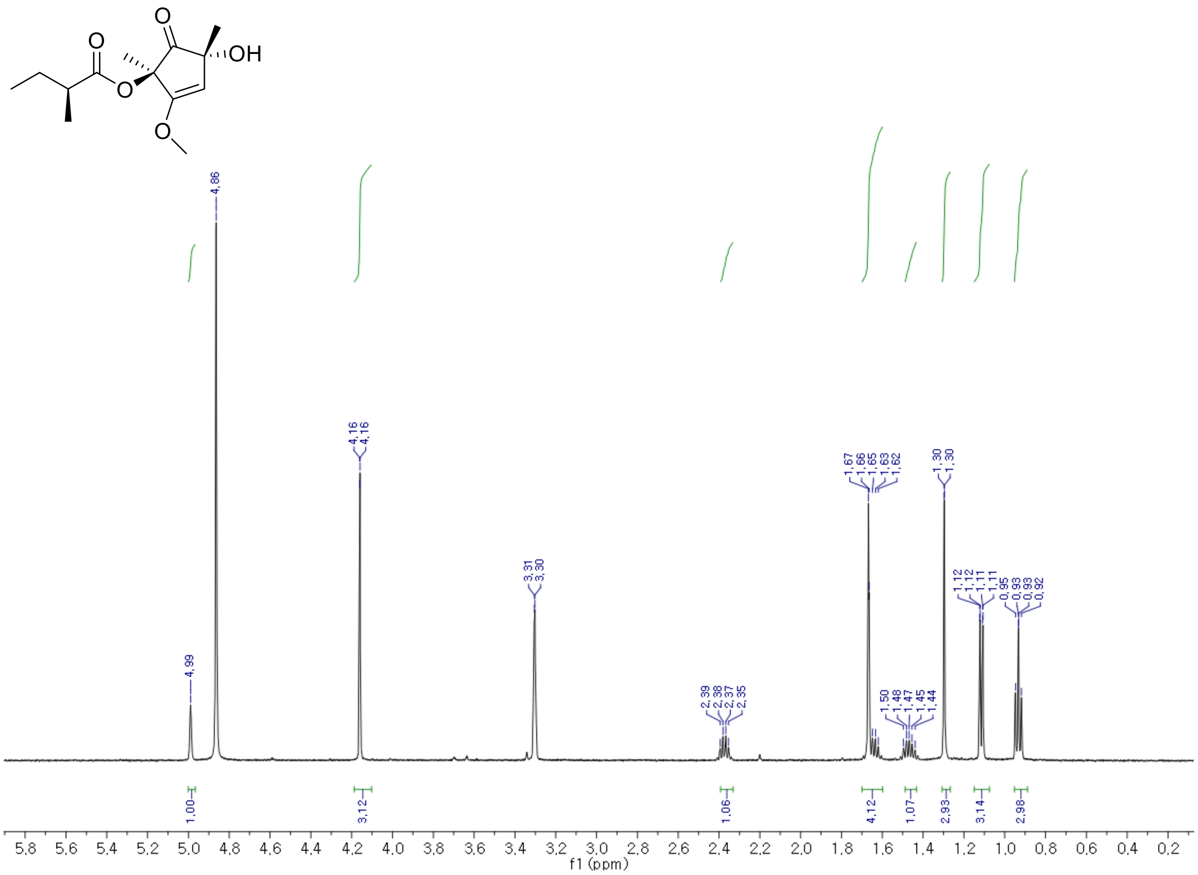

Figure S2. ^{1}H NMR spectrum of deketo-phomaligol A (**1**).

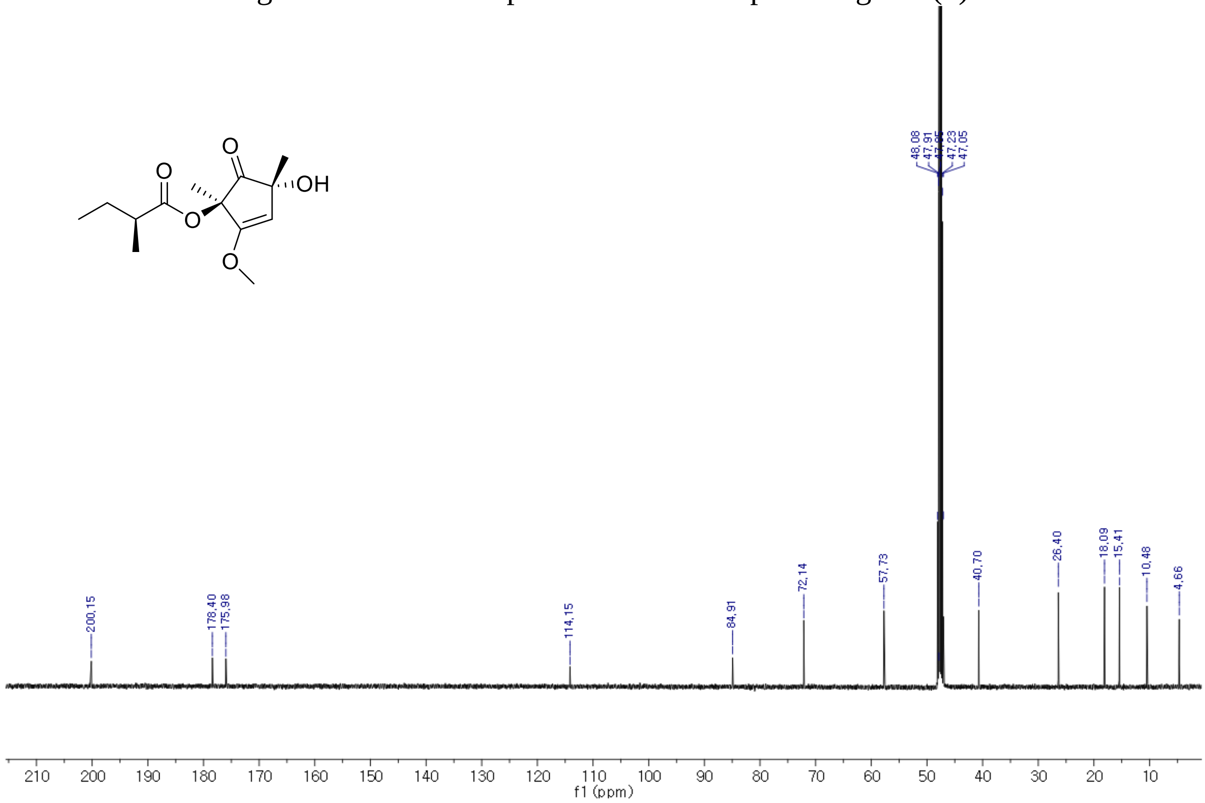

Figure S3. ^{13}C NMR spectrum of deketo-phomaligol A (**1**).

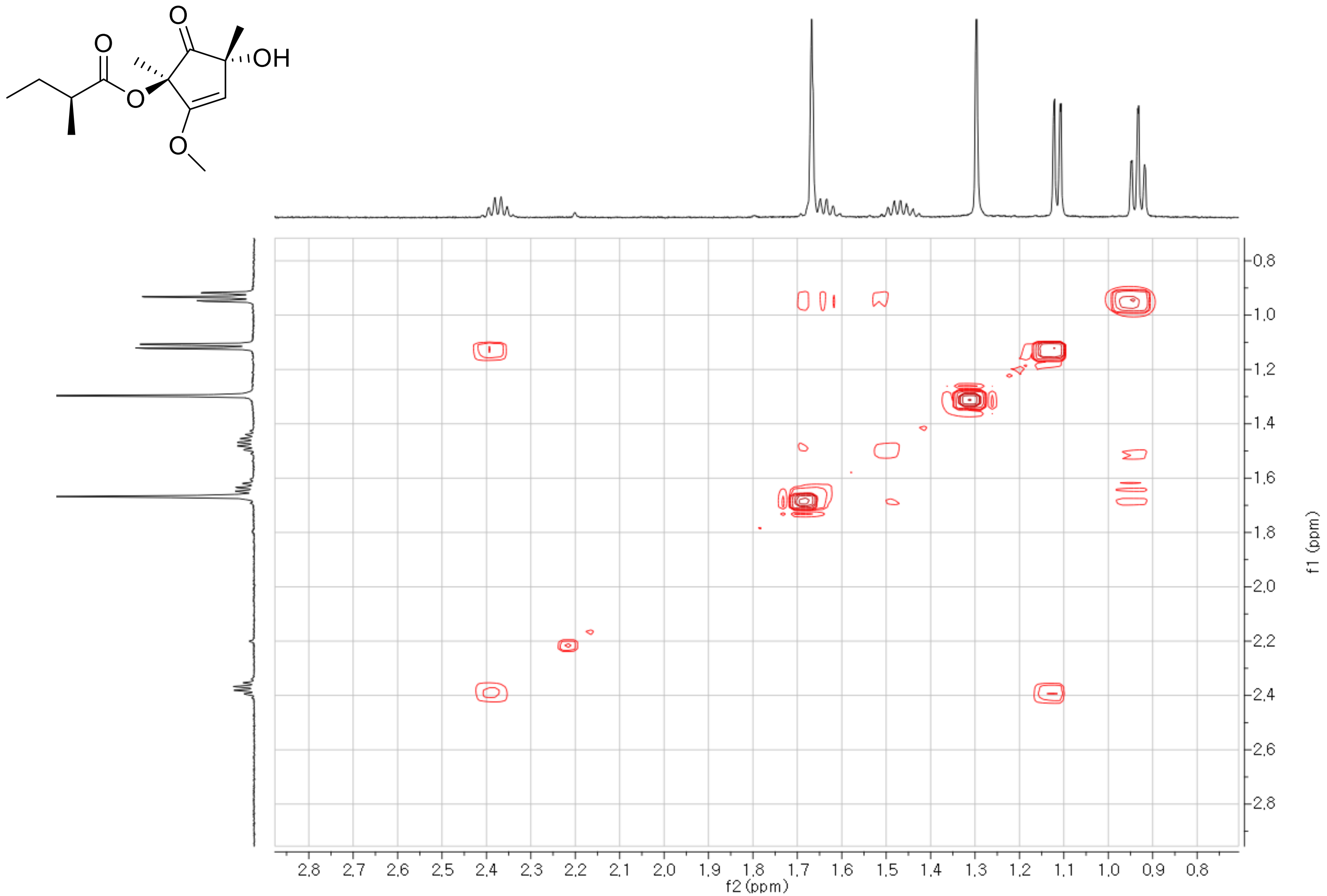

Figure S4. ^{1}H-^{1}H COSY spectrum of deketo-phomaligol A (**1**).

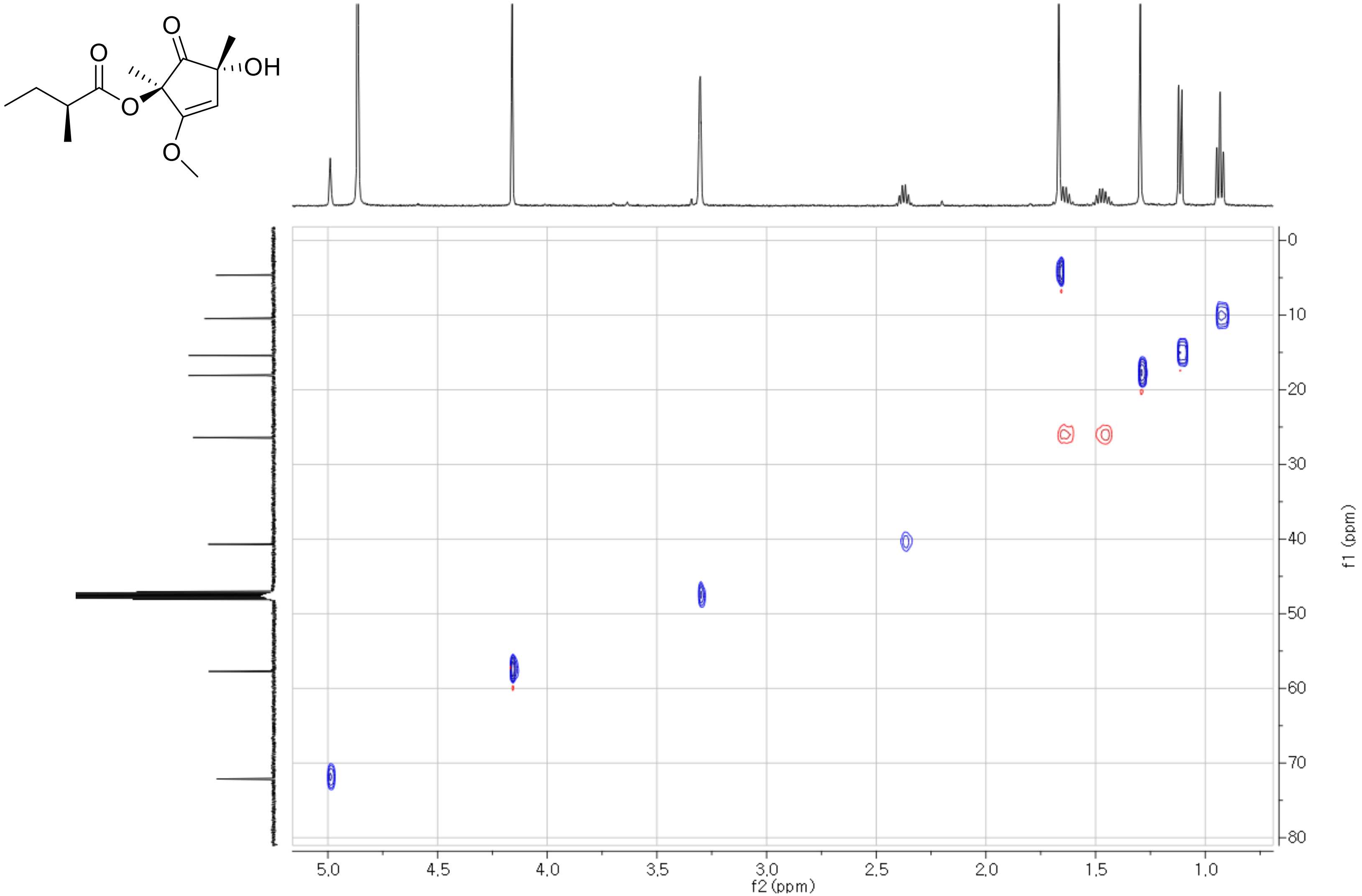

Figure S5. HSQC spectrum of deketo-phomaligol A (**1**).

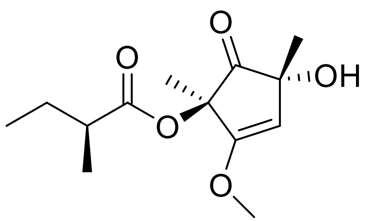

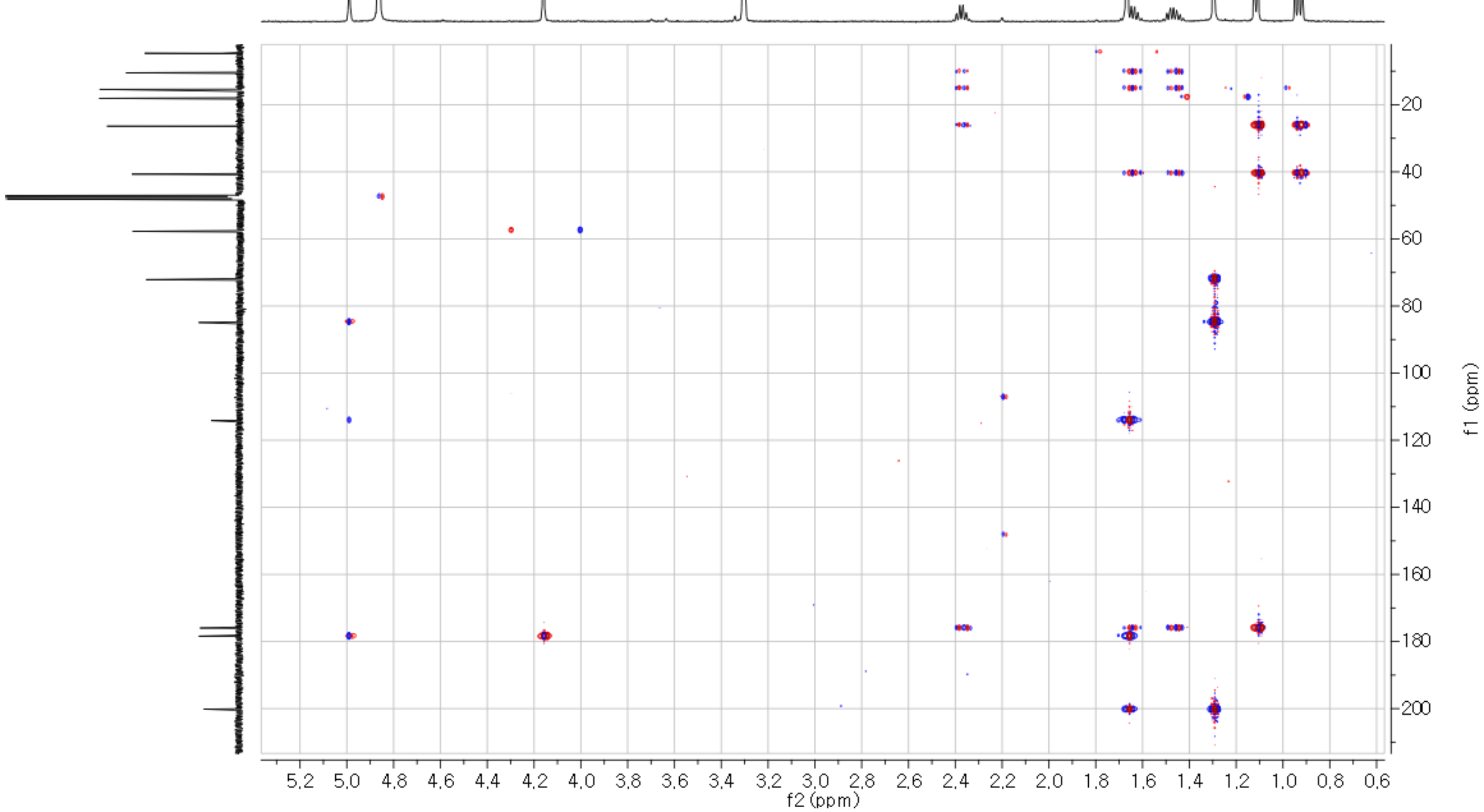

Figure S6. HMBC spectrum of deketo-phomaligol A (**1**).

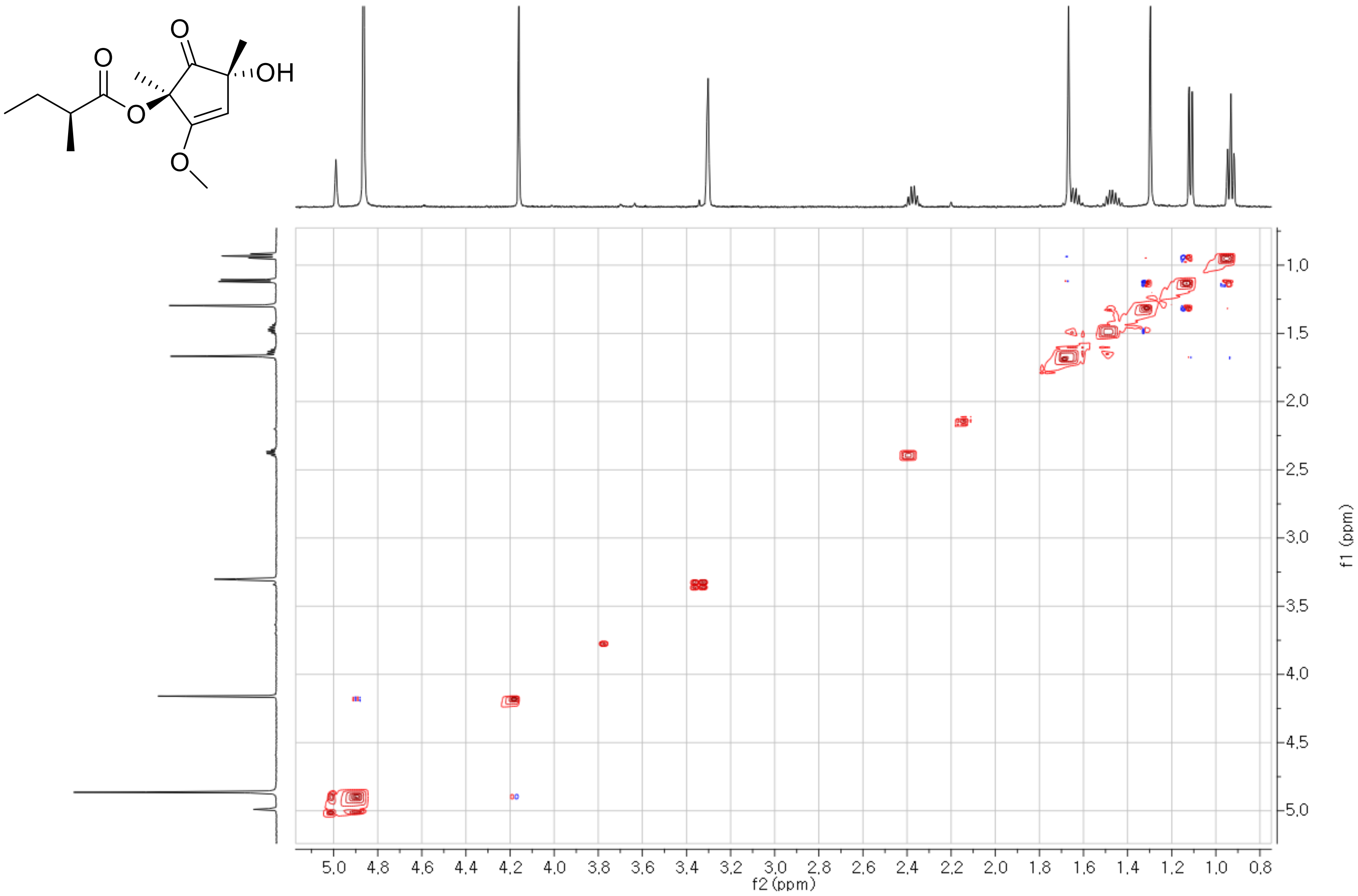

Figure S7. ROESY spectrum of deketo-phomaligol A (**1**).

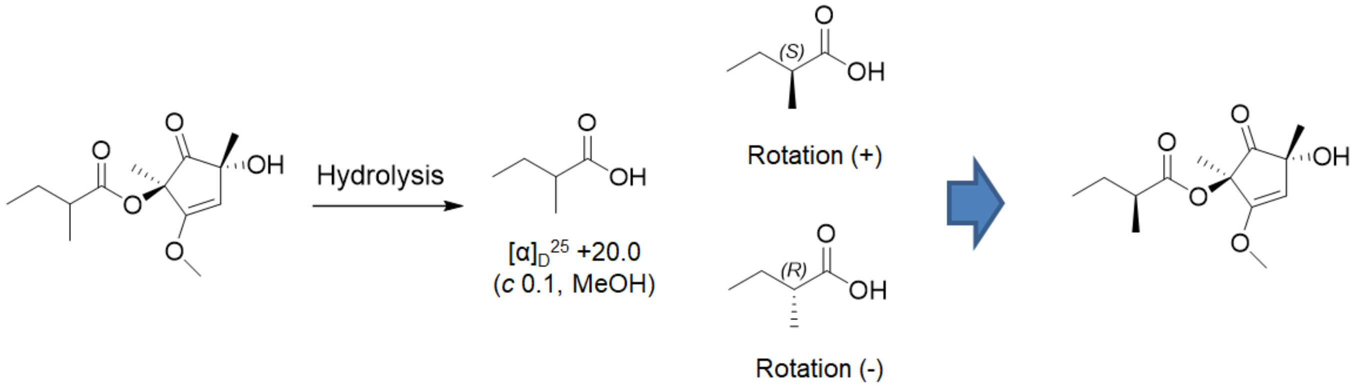

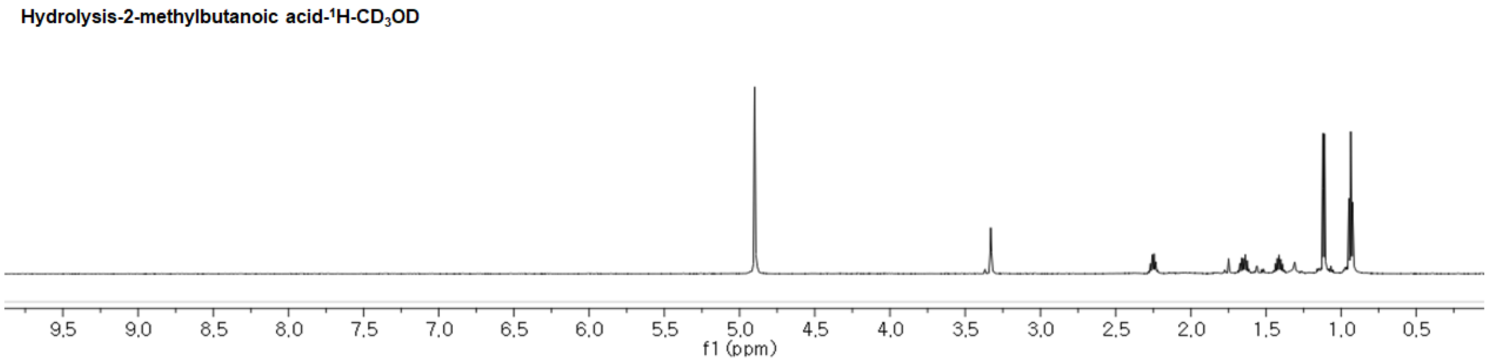

Figure S8. ^{1}H NMR spectrum of 2-methylbutanoic acid by hydrolysis of **1.**

Elemental Composition Report

Single Mass Analysis
Tolerance = 10.0 PPM / DBE: min = -1.5, max = 50.0
Element prediction: Off
Number of isotope peaks used for i-FIT = 3

Monoisotopic Mass, Even Electron Ions
45 formula(e) evaluated with 1 results within limits (up to 50 closest results for each mass)
Elements Used:
C: 0-15 H: 0-30 O: 0-10 Na: 0-1

Minimum:				-1.5				
Maximum:		5.0	10.0	50.0				
Mass	Calc. Mass	mDa	PPM	DBE	i-FIT	Norm	Conf(%)	Formula
209.0791	209.0790	0.1	0.5	2.5	1654.2	n/a	n/a	C9 H14 O4 Na

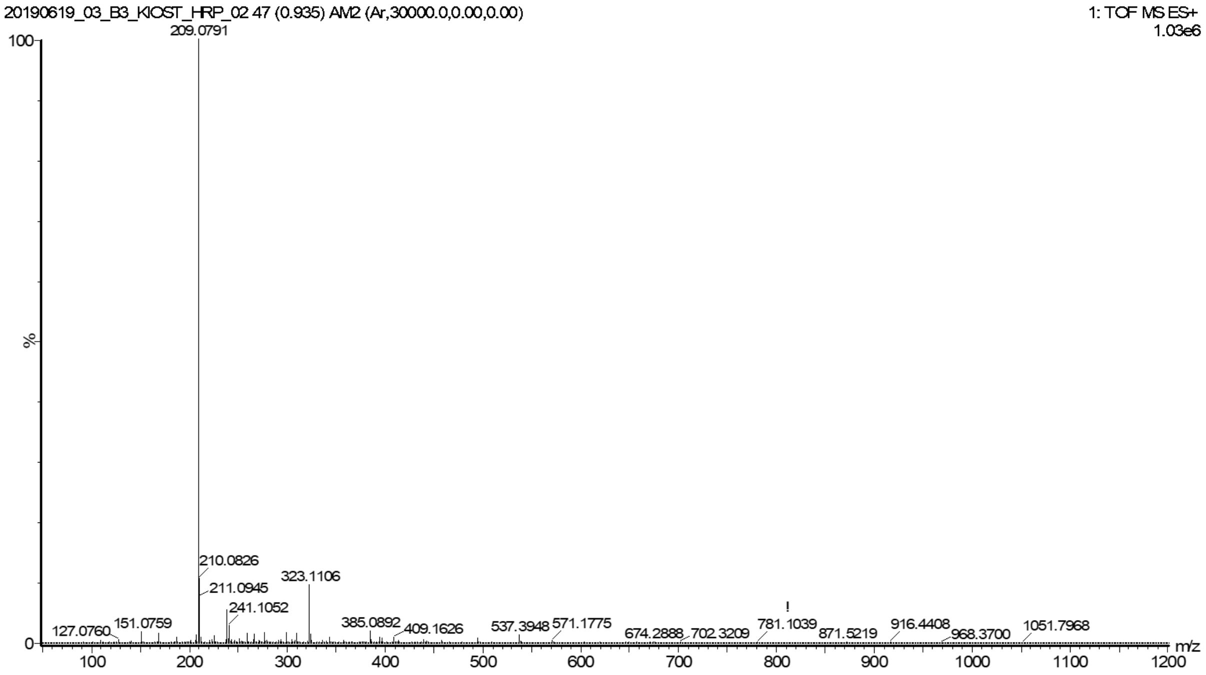

Figure S9. HRESIMS data of phomaligol E (**2**).

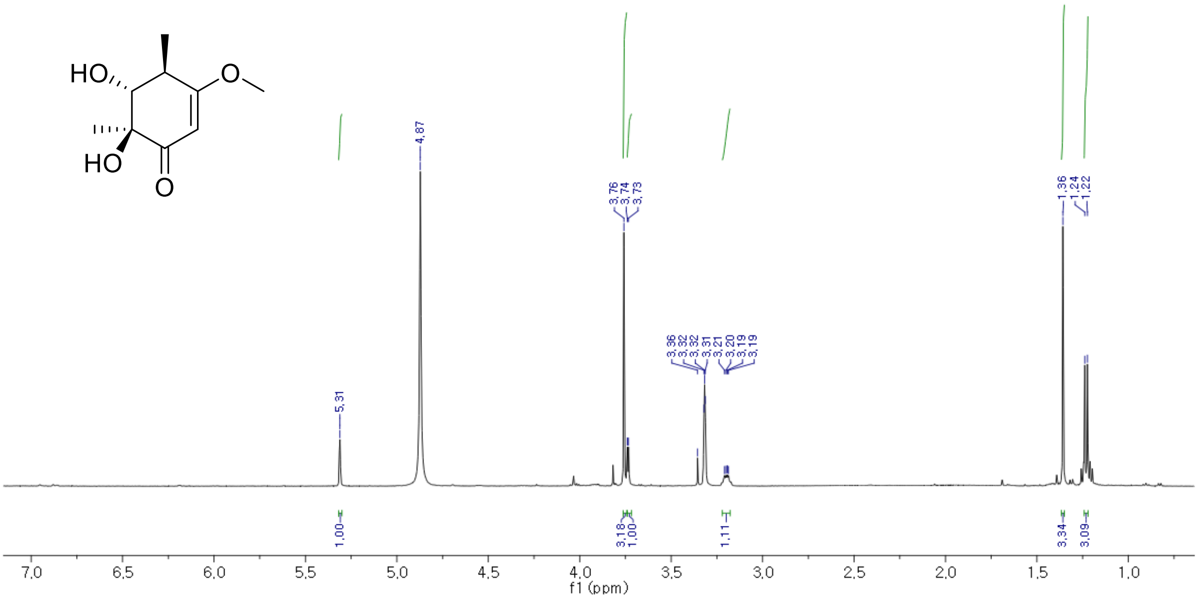

Figure S10. ^{1}H NMR spectrum of phomaligol E (**2**).

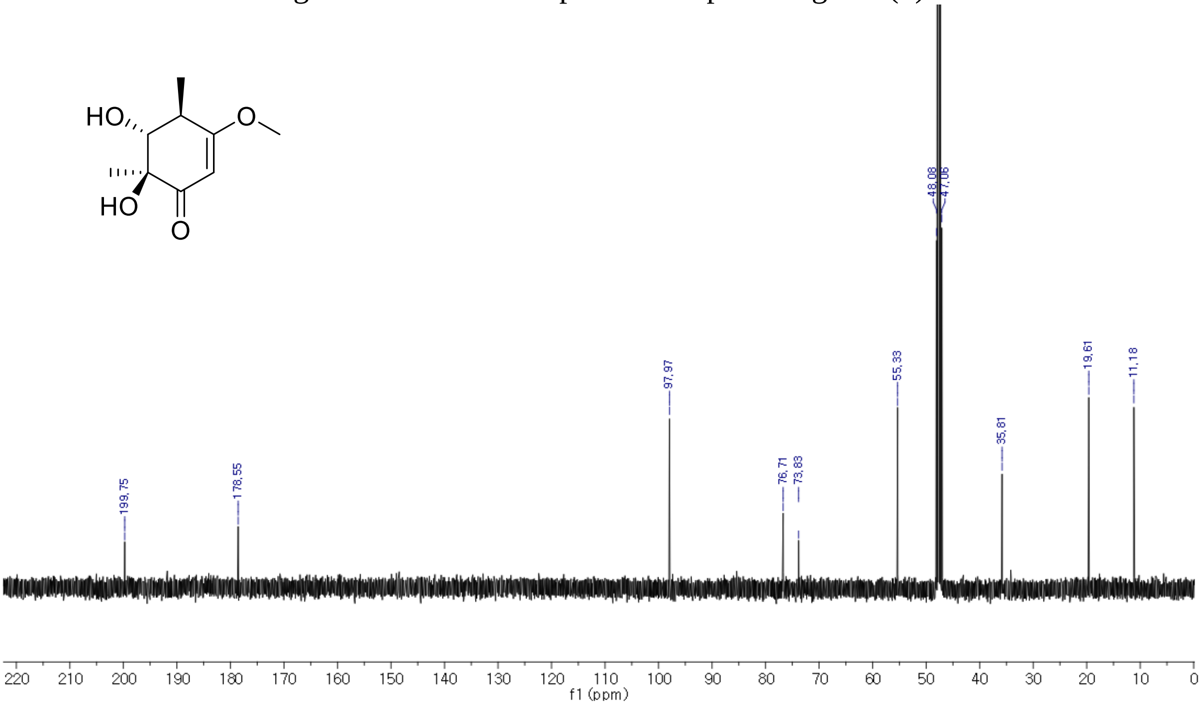

Figure S11. ^{13}C NMR spectrum of phomaligol E (**2**).

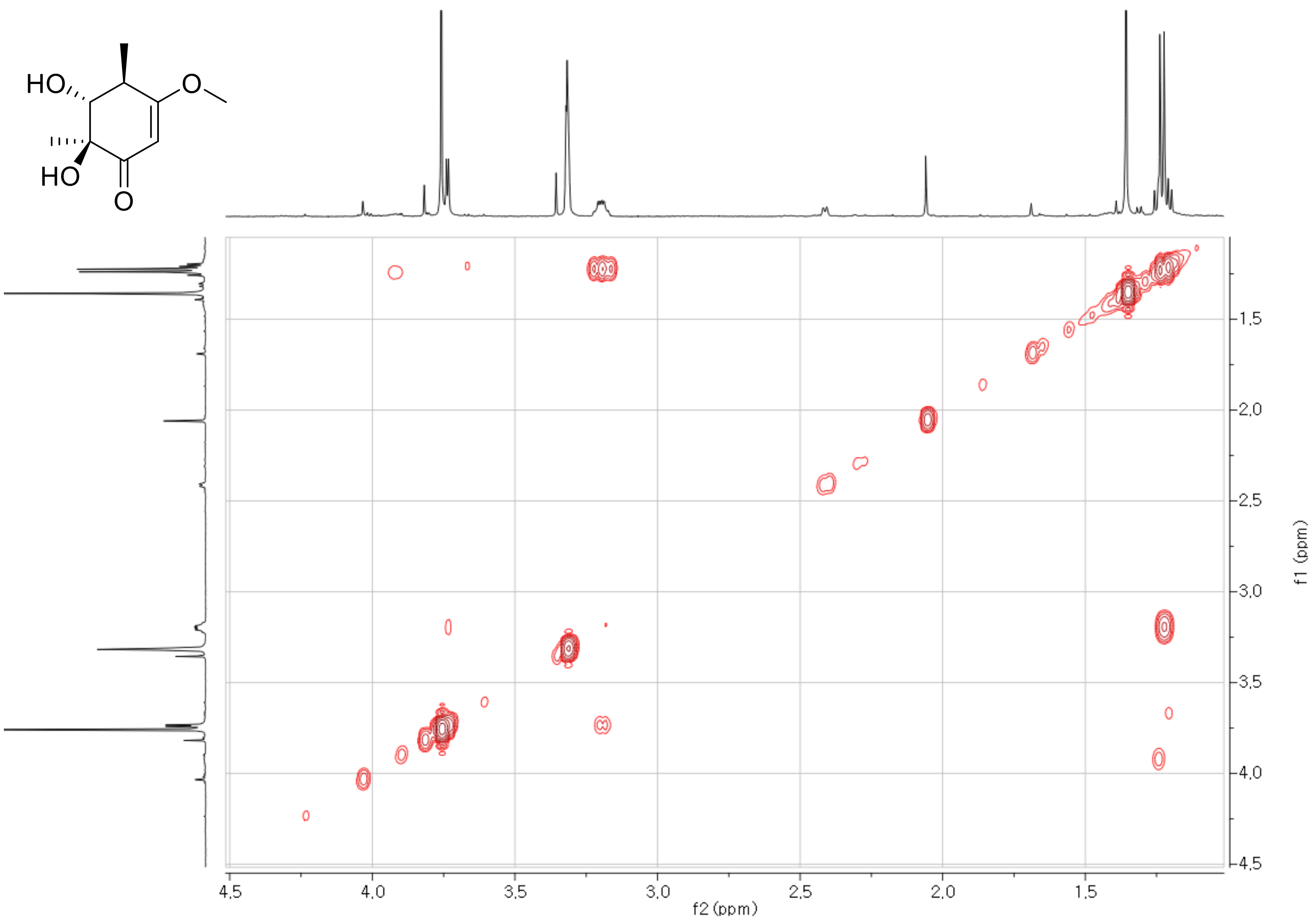

Figure S12. ^{1}H-^{1}H COSY spectrum of phomaligol E (**2**).

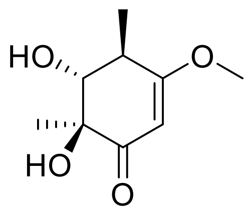

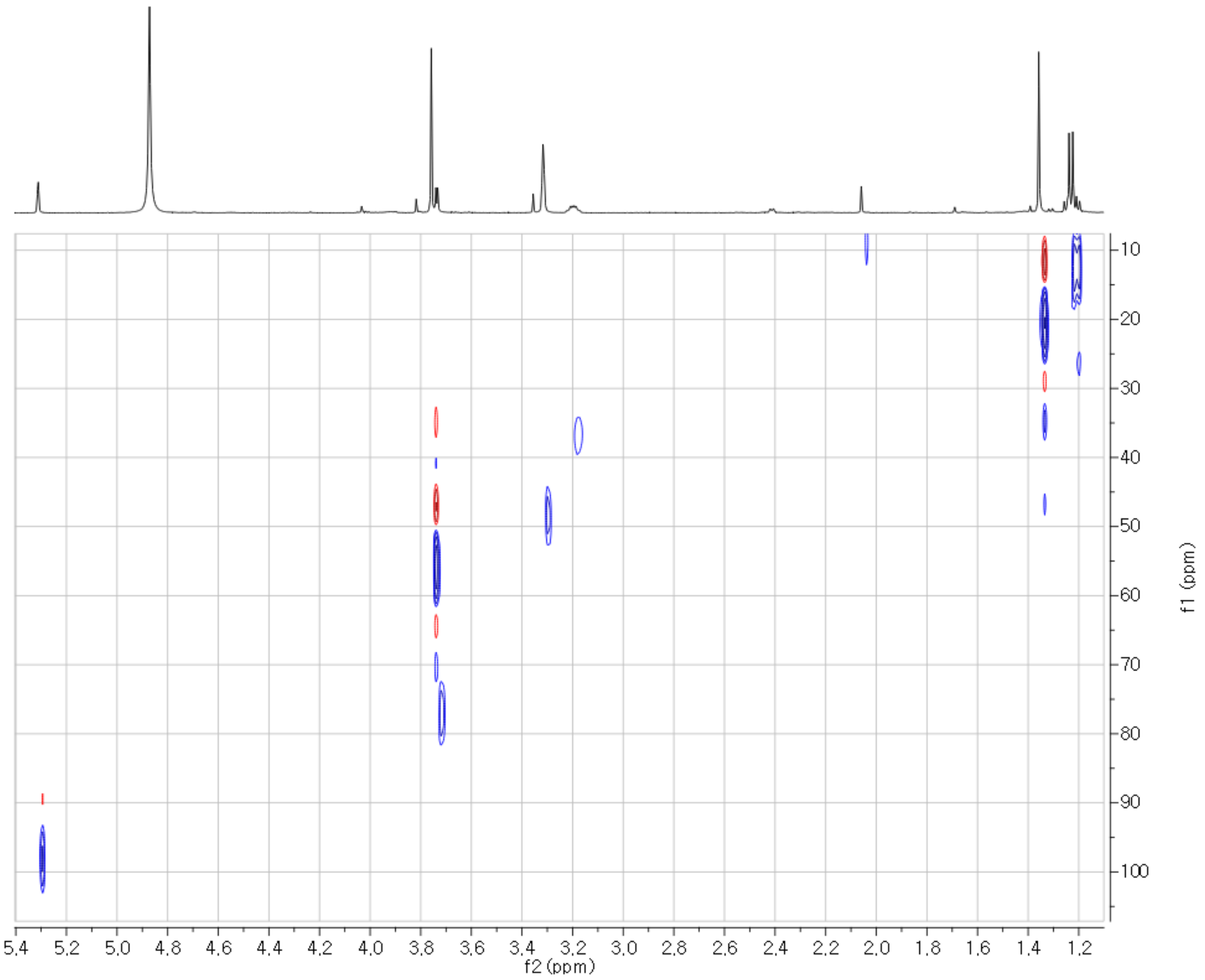

Figure S13. HSQC spectrum of phomaligol E (**2**).

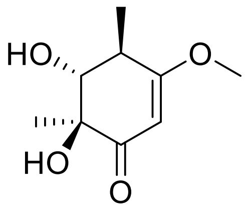

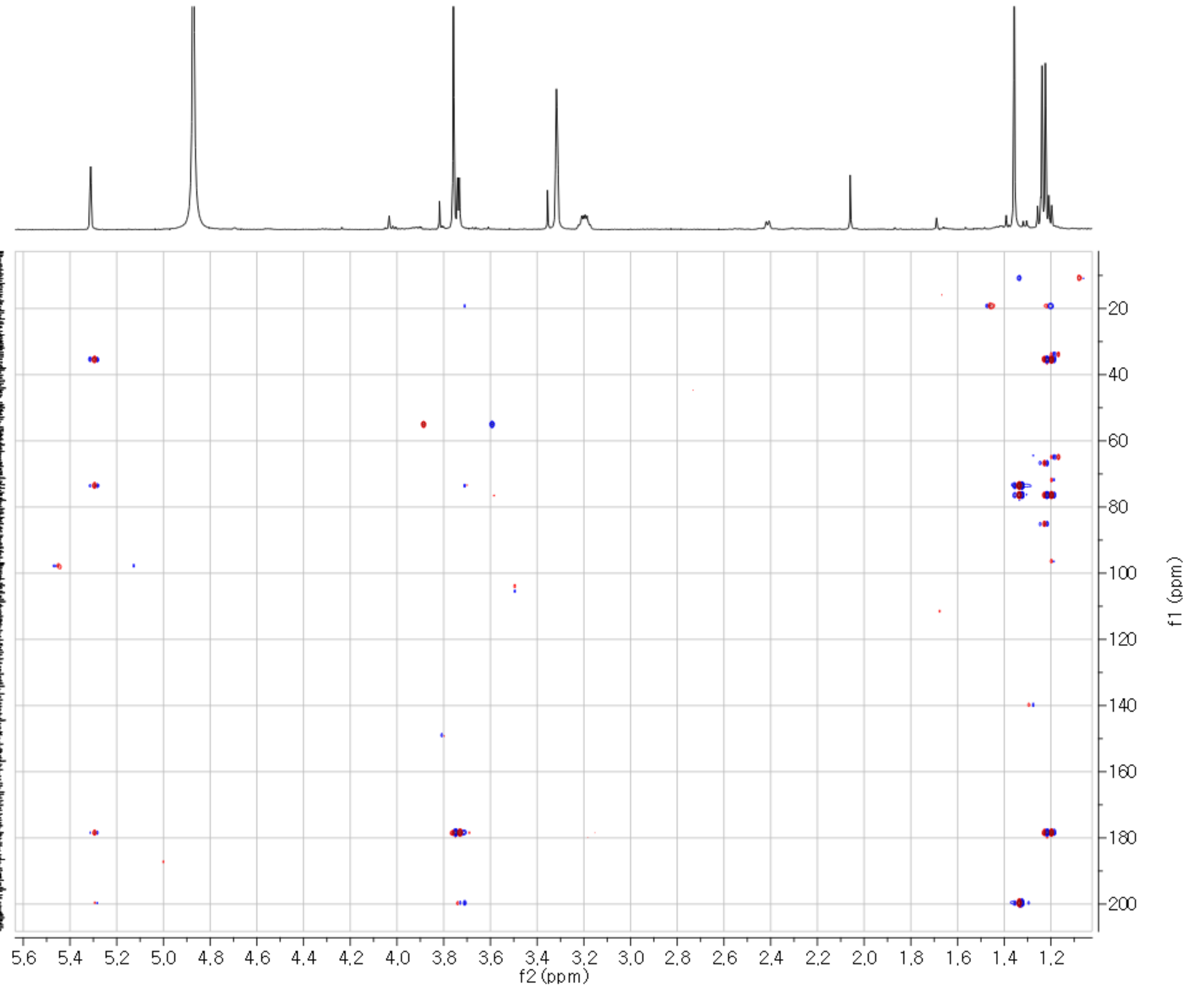

Figure S14. HMBC spectrum of phomaligol E (**2**).

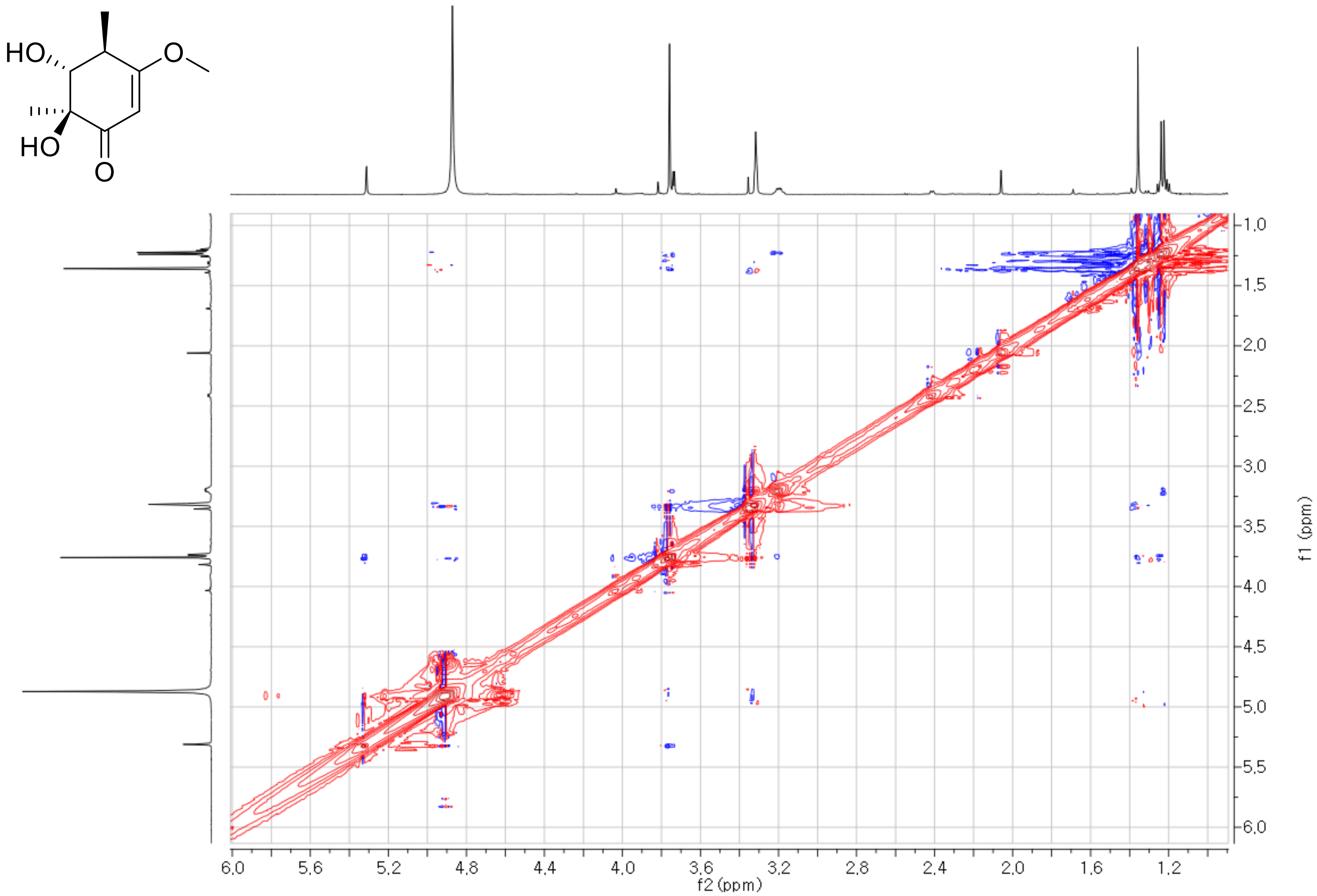

Figure S15. ROESY spectrum of phomaligol E (**2**).

Elemental Composition Report

Single Mass Analysis
Tolerance = 10.0 PPM / DBE: min = -1.5, max = 50.0
Element prediction: Off
Number of isotope peaks used for i-FIT = 3

Monoisotopic Mass, Even Electron Ions
42 formula(e) evaluated with 2 results within limits (up to 50 closest results for each mass)
Elements Used:
C: 0-15 H: 0-30 O: 0-10 Na: 0-1

Minimum:				-1.5				
Maximum:		5.0	10.0	50.0				
Mass	Calc. Mass	mDa	PPM	DBE	i-FIT	Norm	Conf(%)	Formula
249.1104	249.1103	0.1	0.4	3.5	1382.9	1.226	29.35	C12 H18 O4 Na

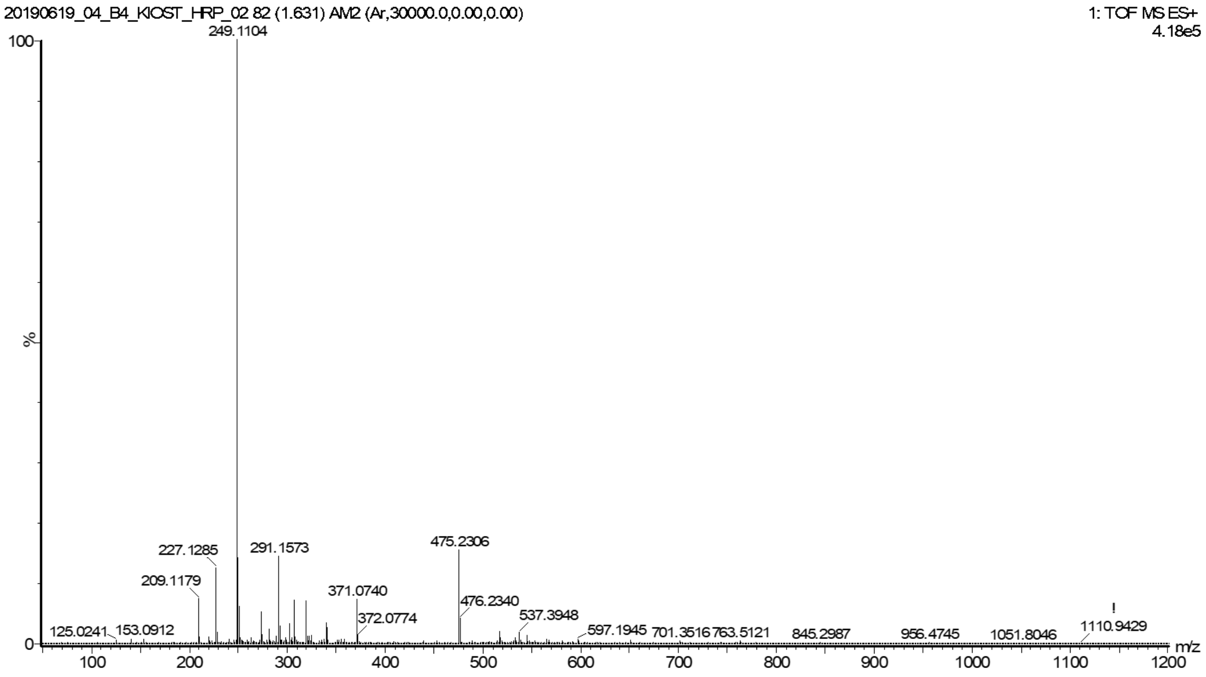

Figure S16. HRESIMS data of sydowione A (**3**).

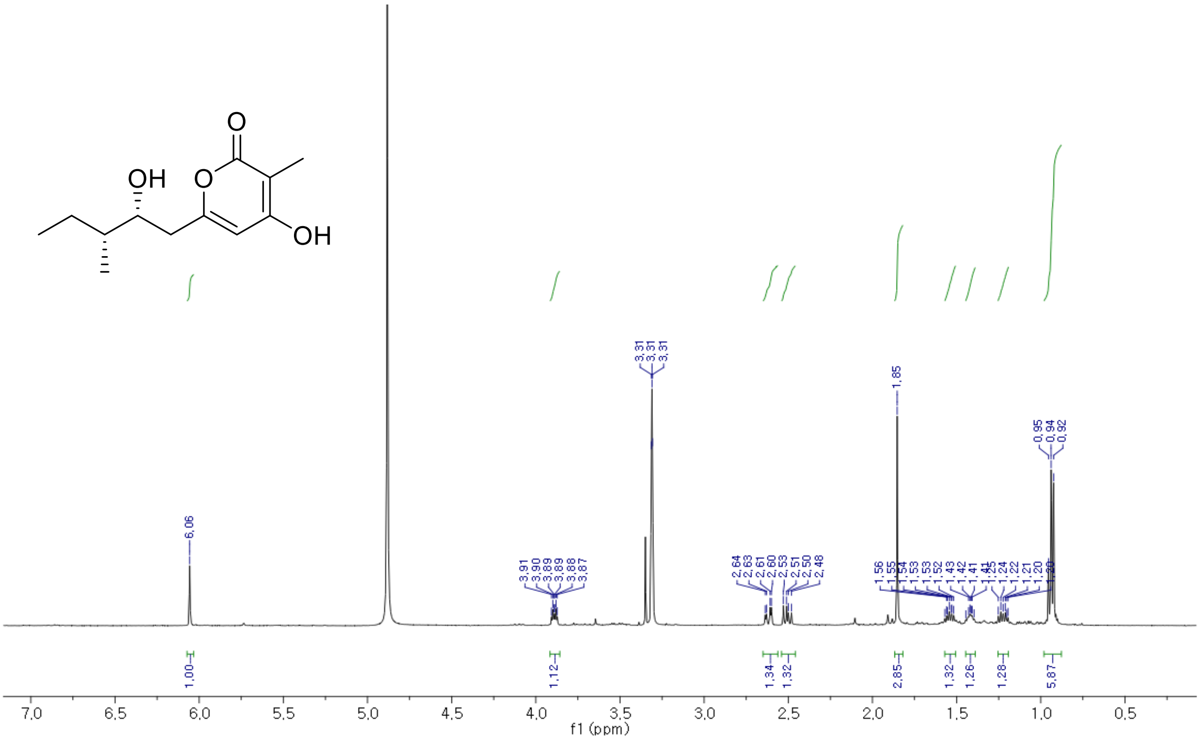

Figure S17. ^{1}H NMR spectrum of sydowione A (**3**).

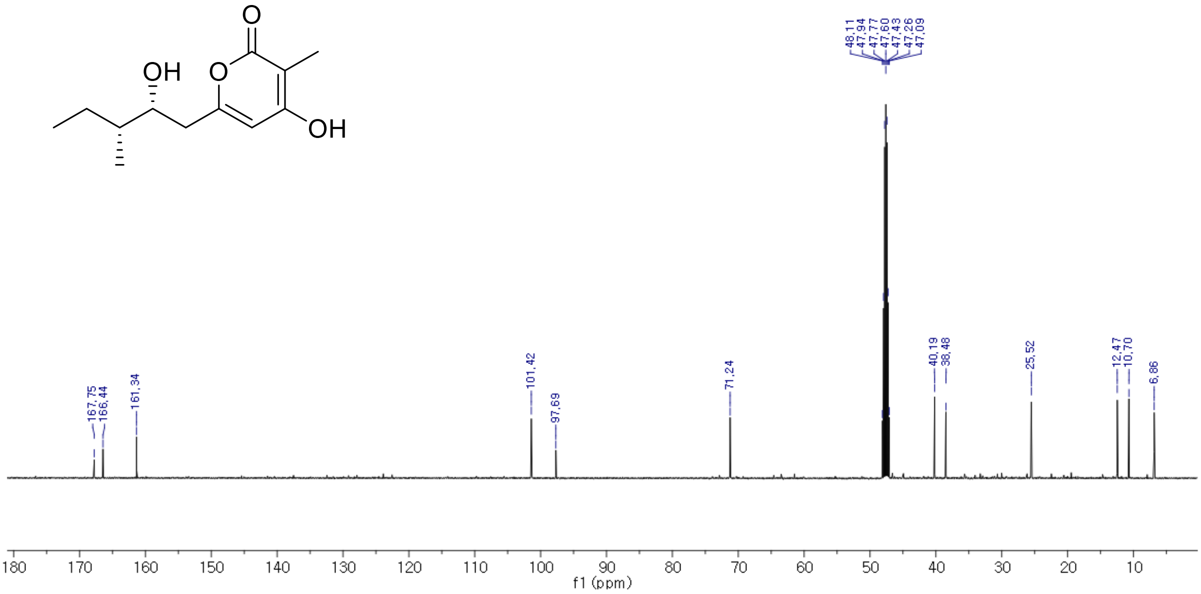

Figure S18. ^{13}C NMR spectrum of sydowione A (**3**).

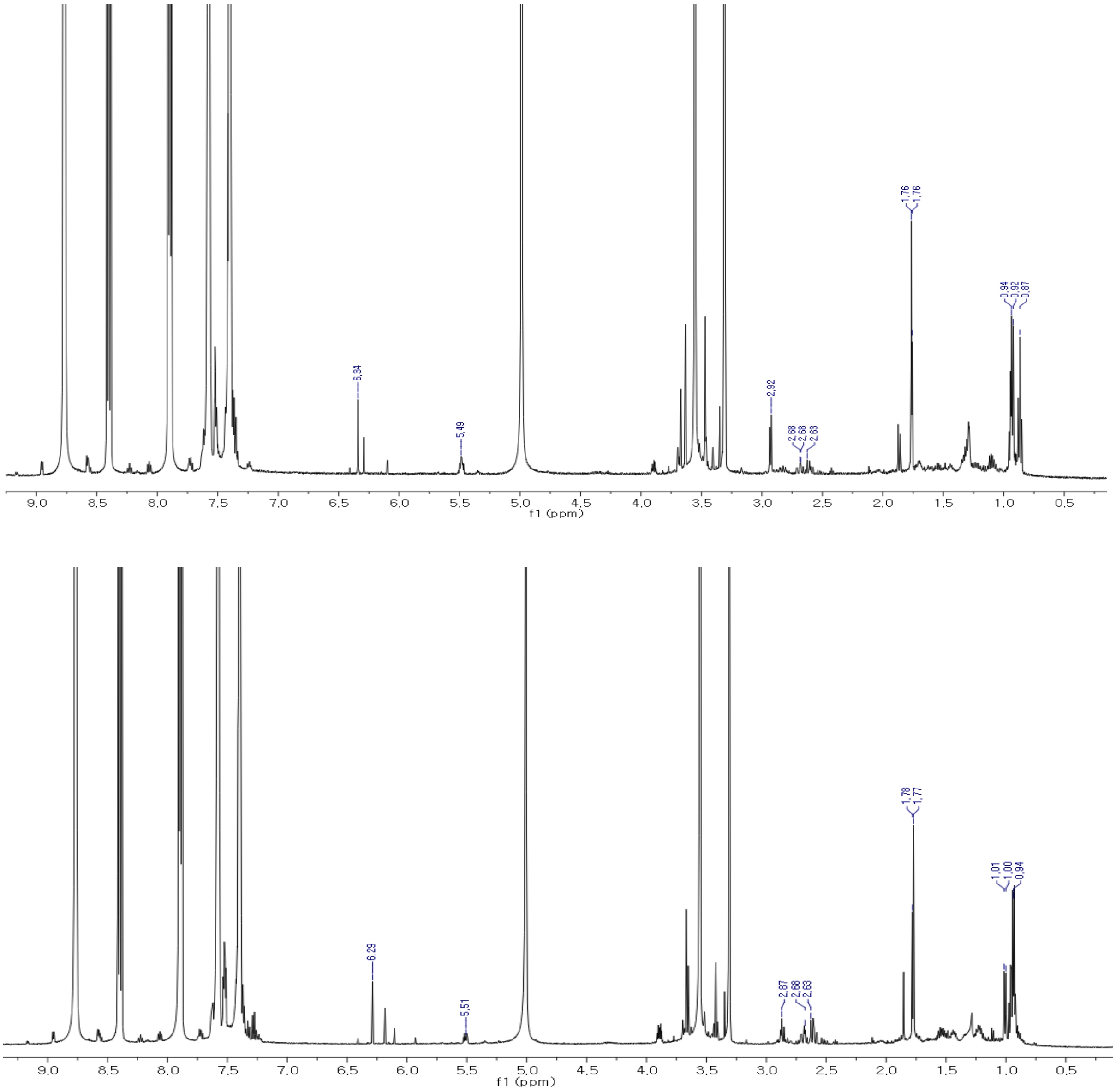

Figure S19. ^{1}H NMR spectrum of (*S*) and (*R*) MTPA (**3a** and **3b**).

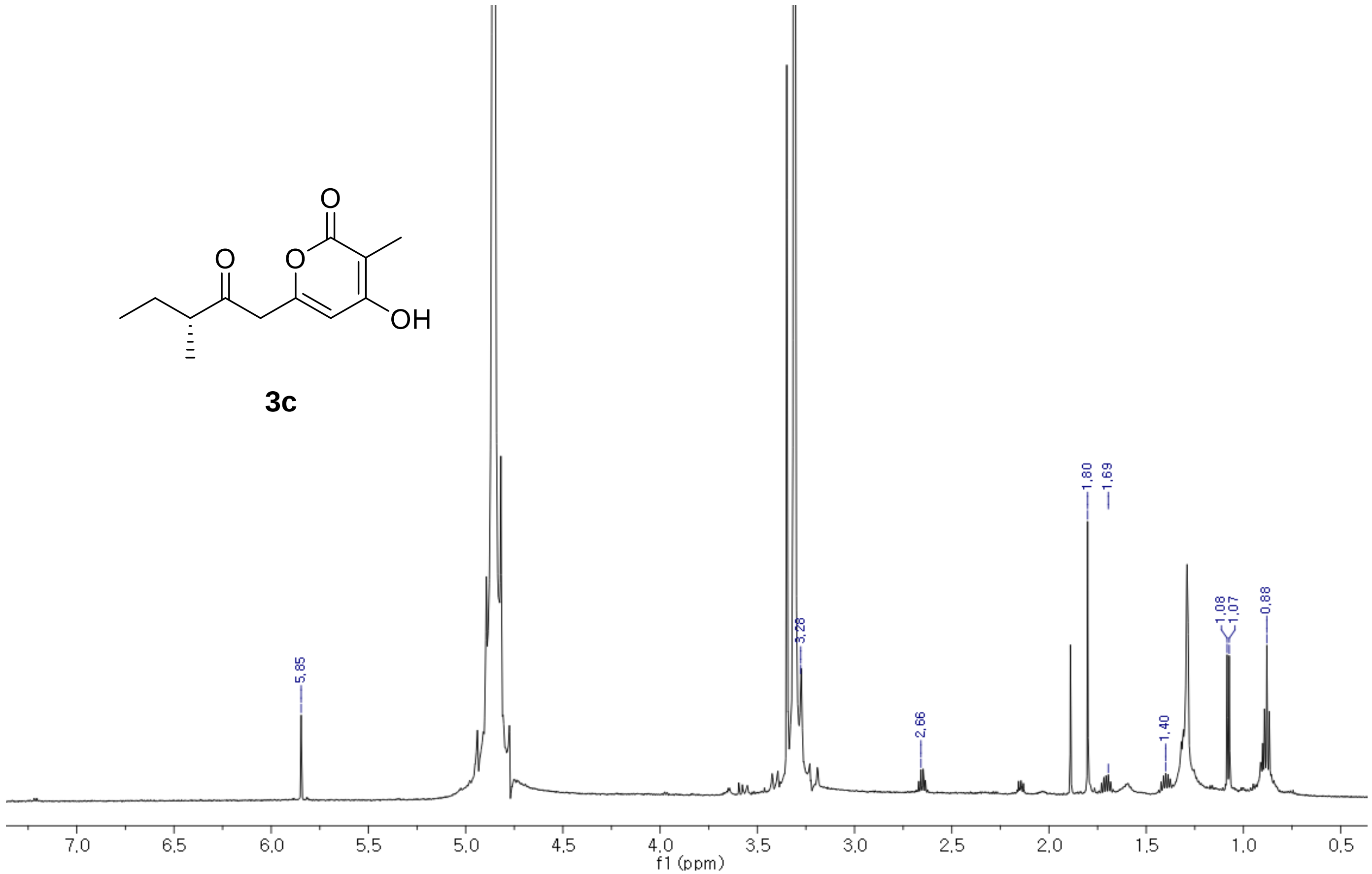

Figure S20. ^{1}H NMR spectrum of oxidation of **3** (**3c**).

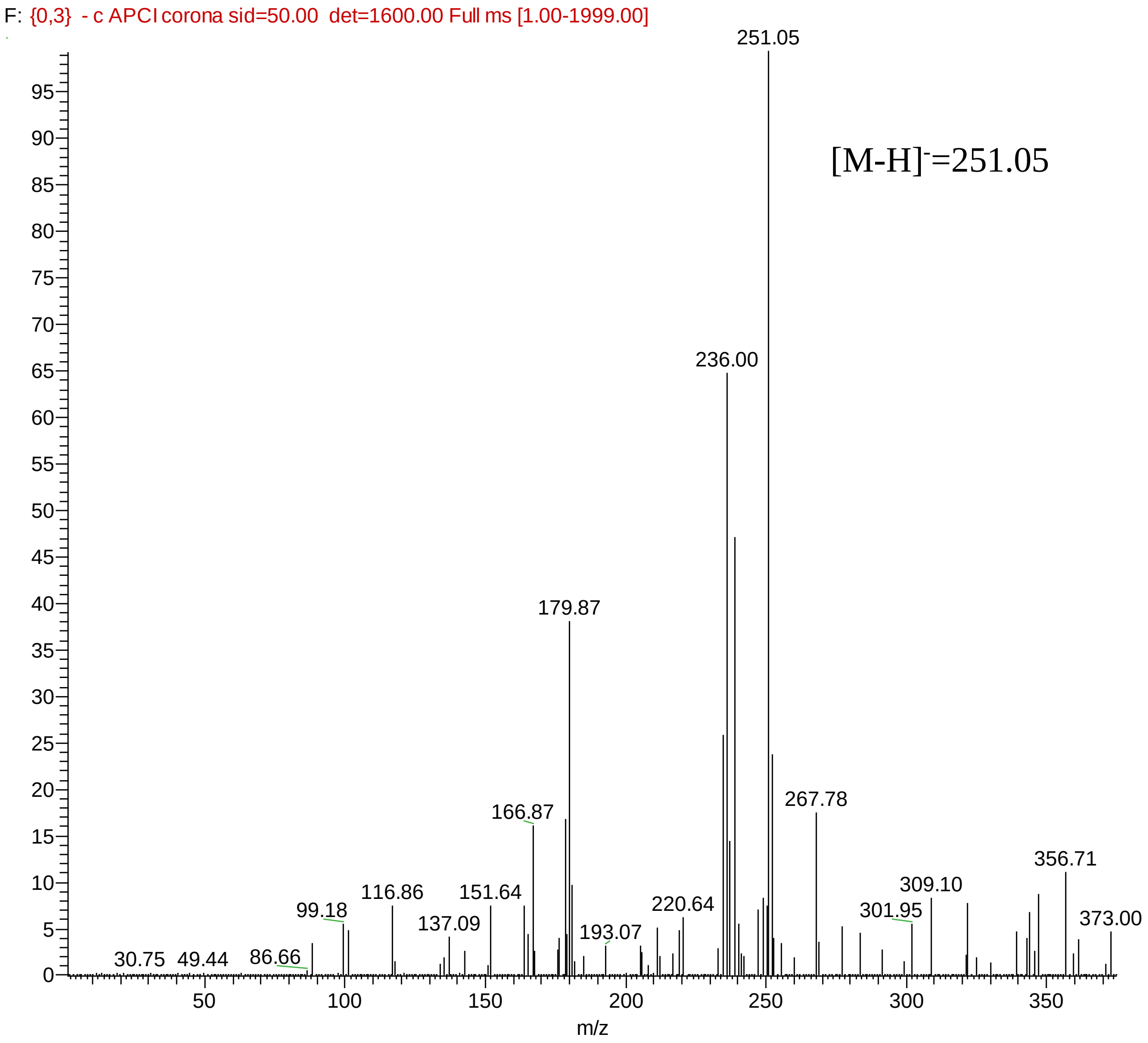

Figure S21. LRMS data of 2,6-dimethyl-3-O -methyl-4-(2-methylbutyryl)phloroglucinol (**4**).

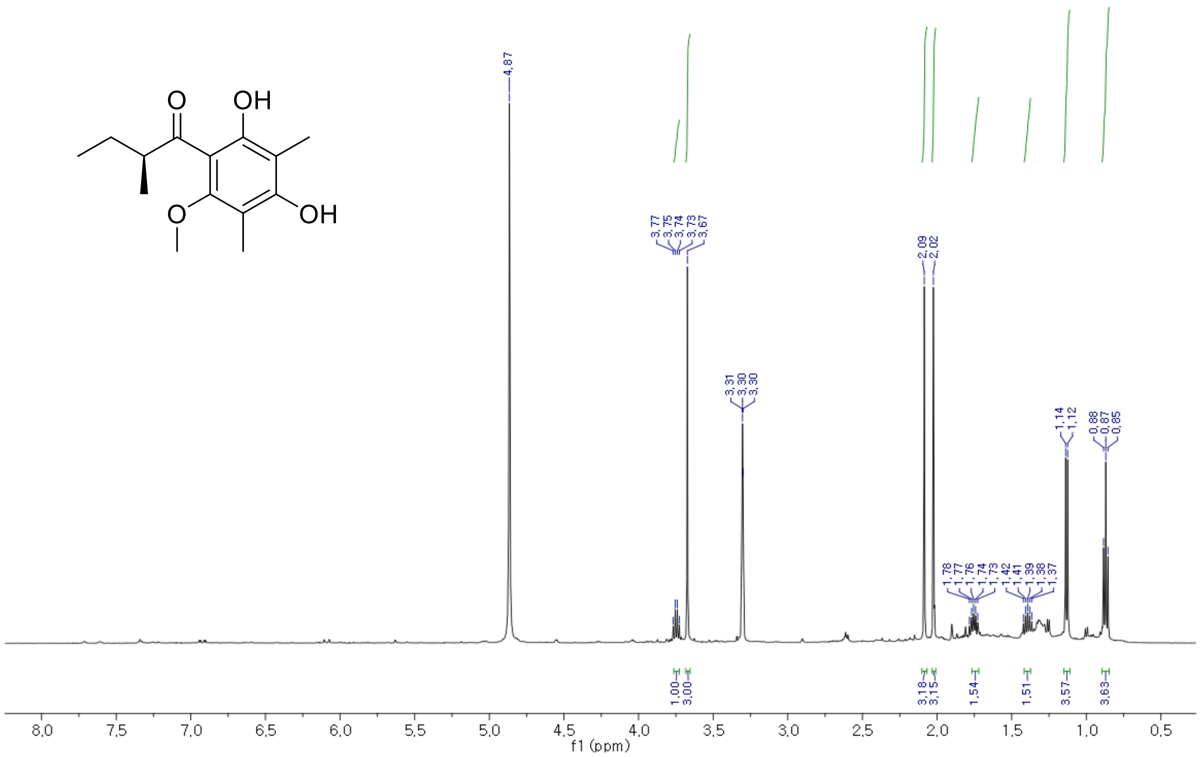

Figure S22. ^{1}H NMR spectrum of 2,6-dimethyl-3-O -methyl-4-(2-methylbutyryl)phloroglucinol (**4**).

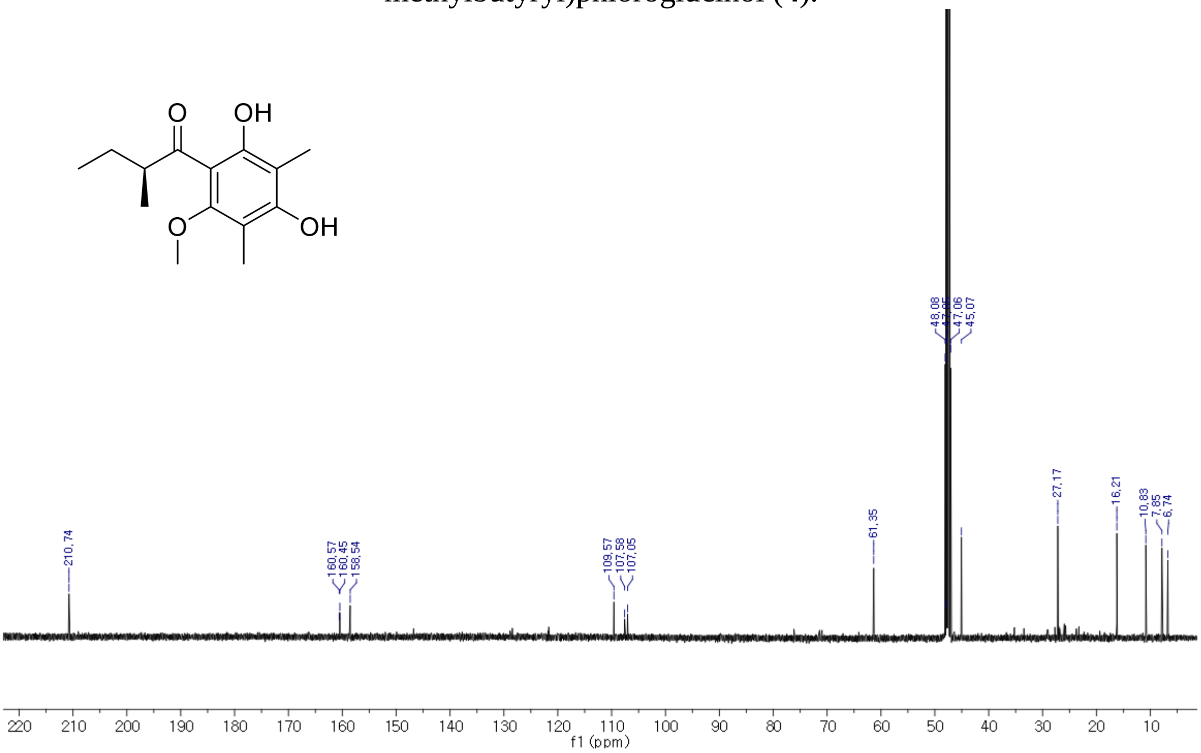

Figure S23. ^{13}C NMR spectrum of 2,6-dimethyl-3-O -methyl-4-(2-methylbutyryl)phloroglucinol (**4**).

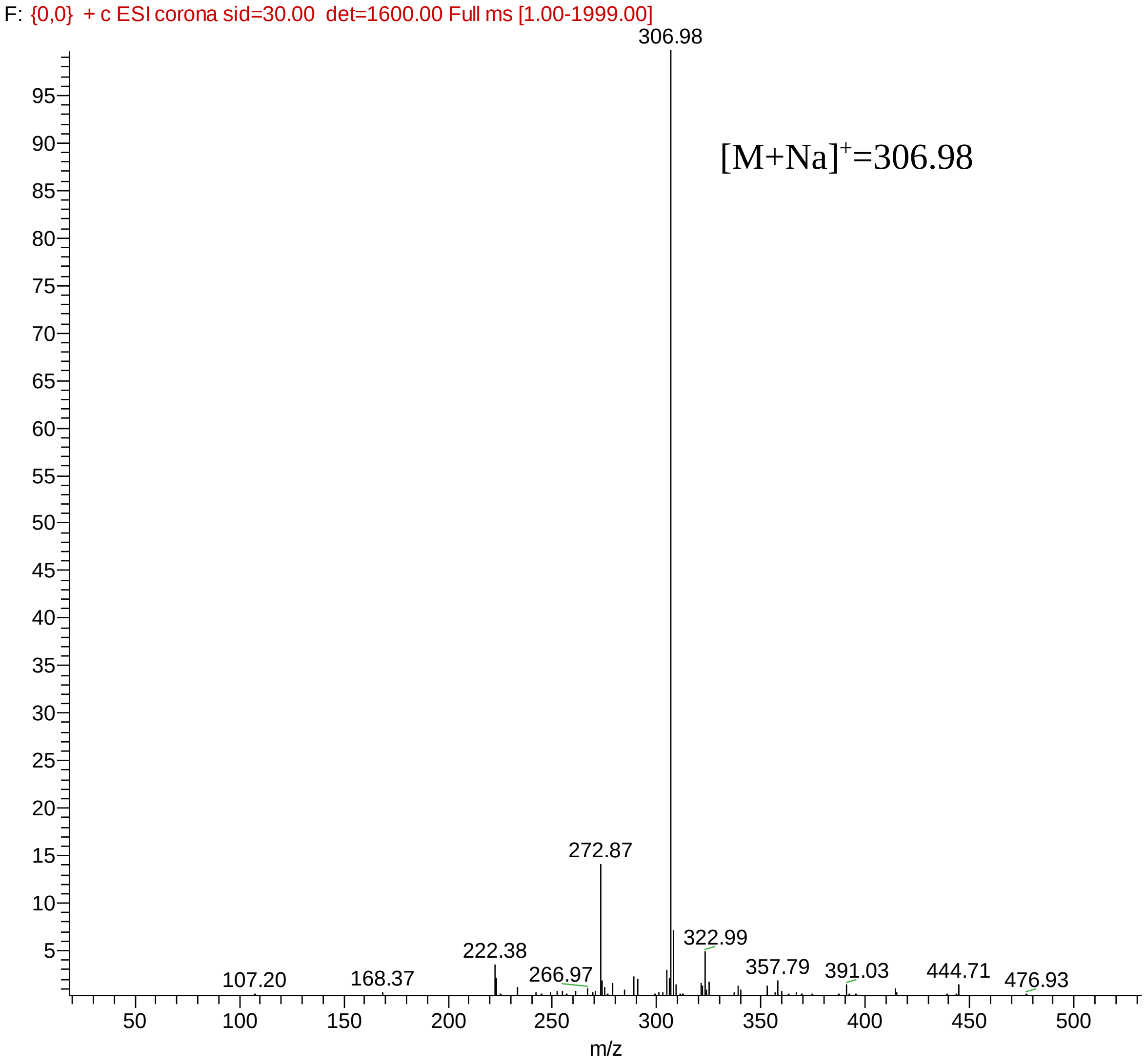

Figure S24. LRMS data of phomaligol A (**5**).

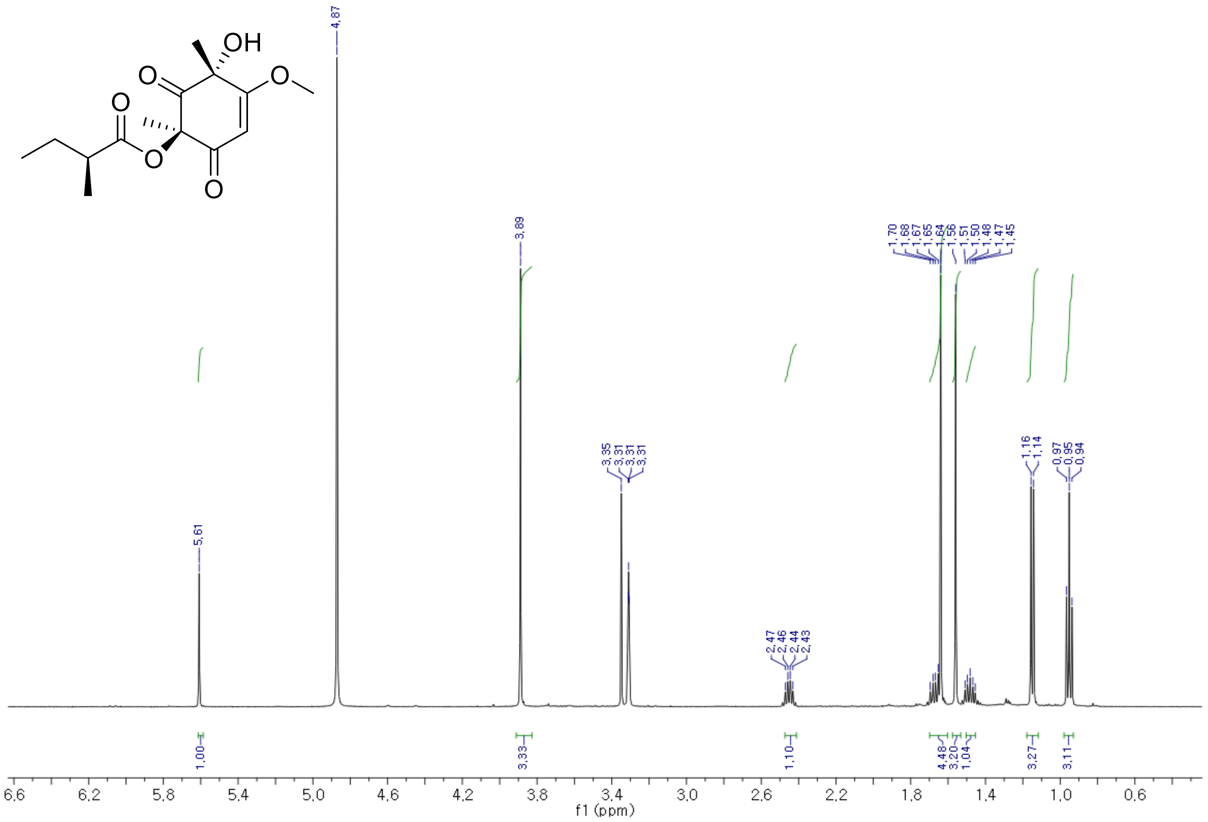

Figure S25. ^{1}H NMR spectrum of phomaligol A (**5**).

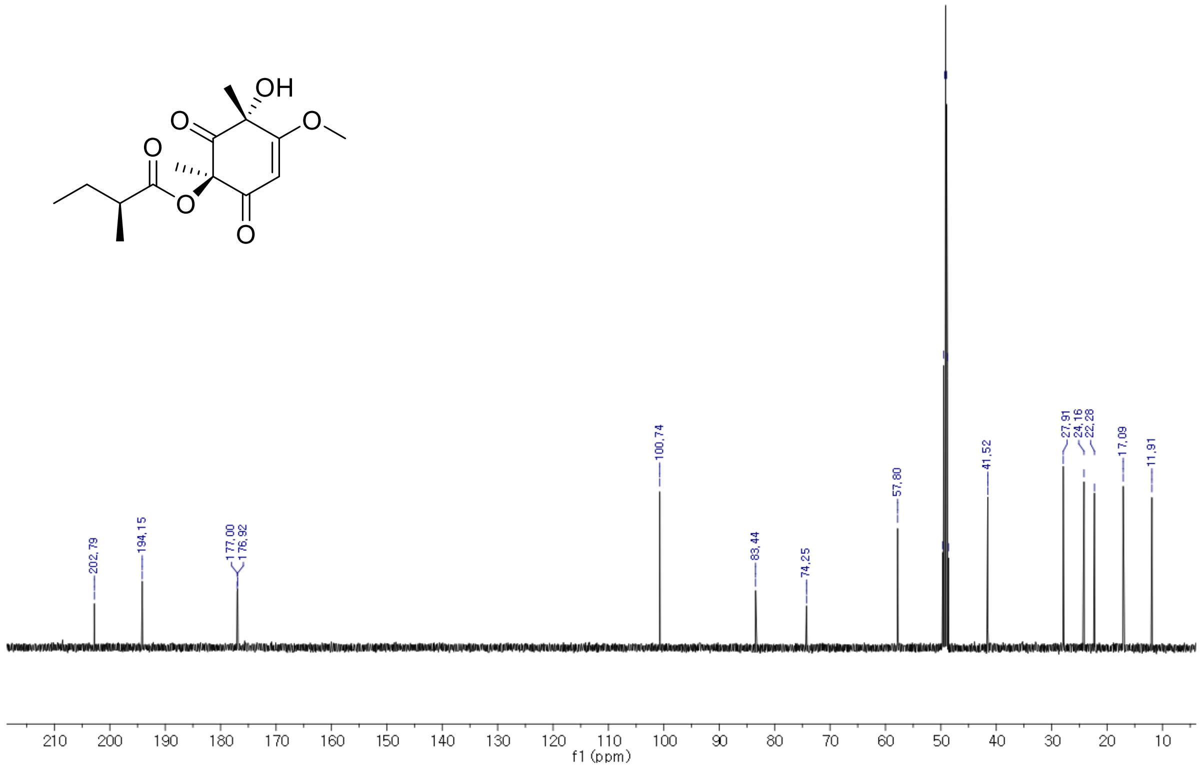

Figure S26. ^{13}C NMR spectrum of phomaligol A (**5**).

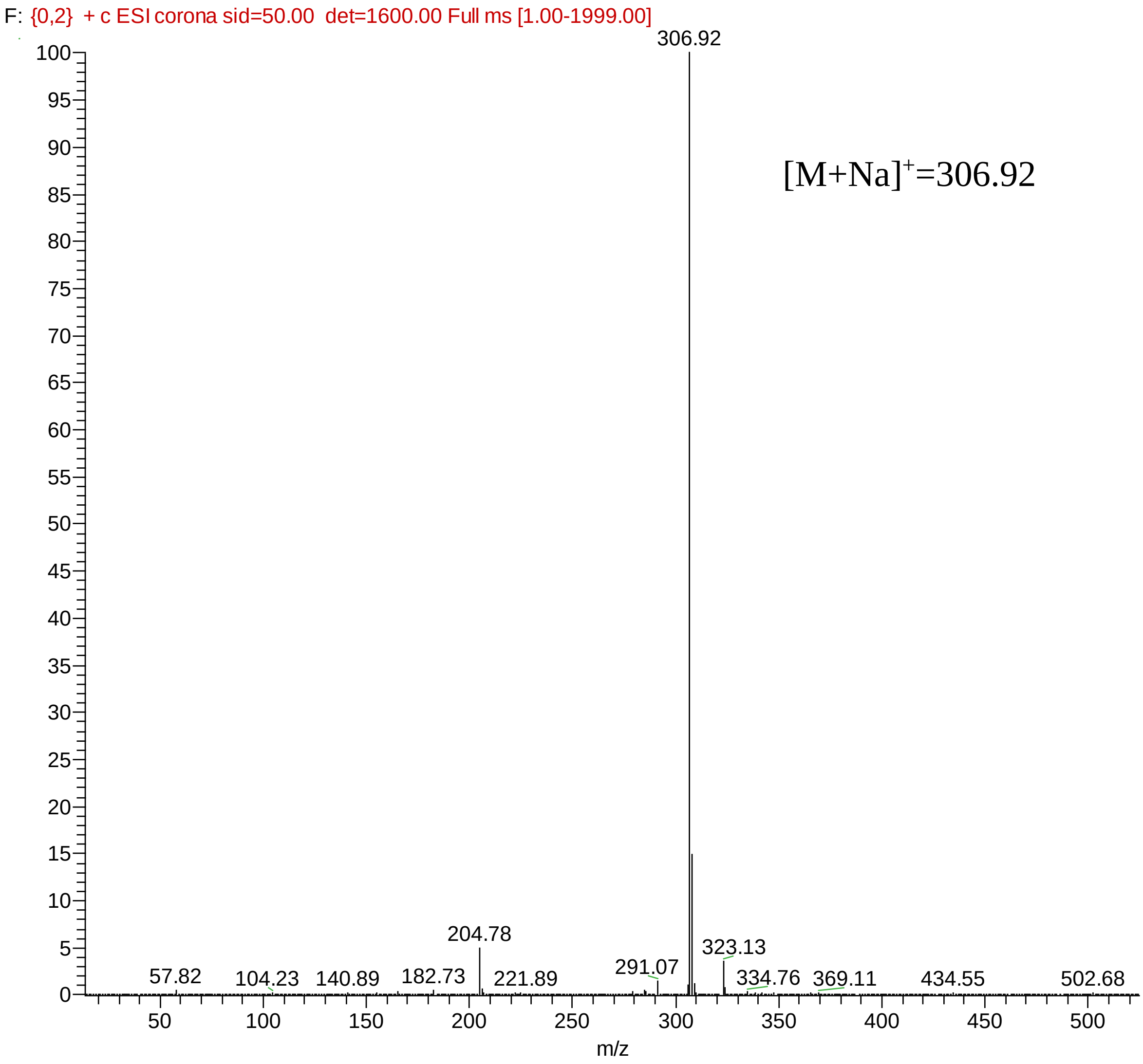

Figure S27. LRMS data of phomaligol A$_1$ (**6**).

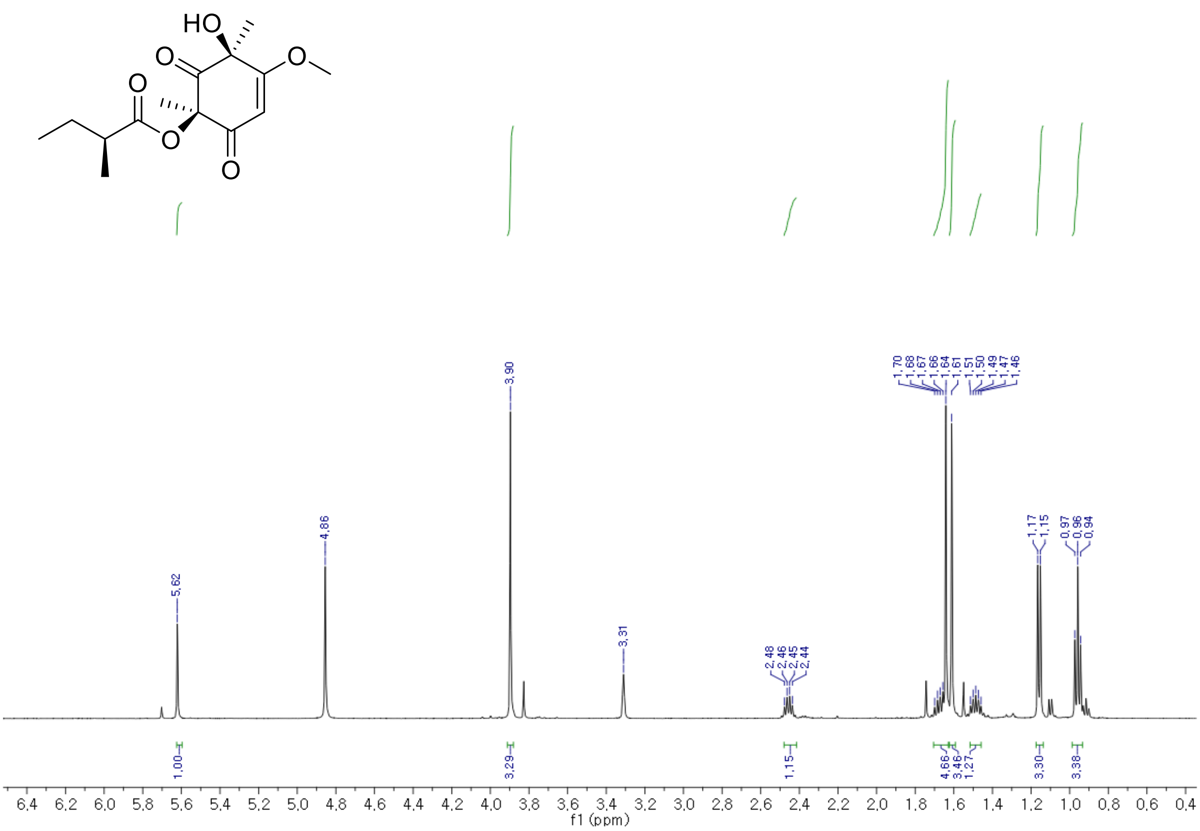

Figure S28. ^{1}H NMR spectrum of phomaligol A_1 (**6**).

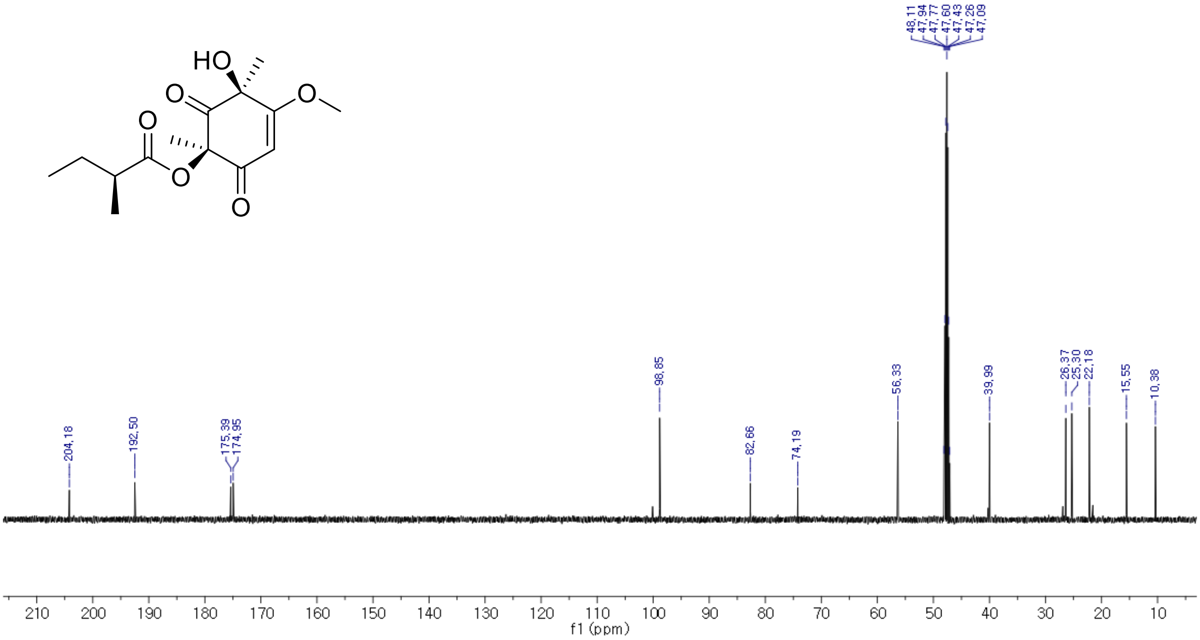

Figure S29. ^{13}C NMR spectrum of phomaligol A_1 (**6**).

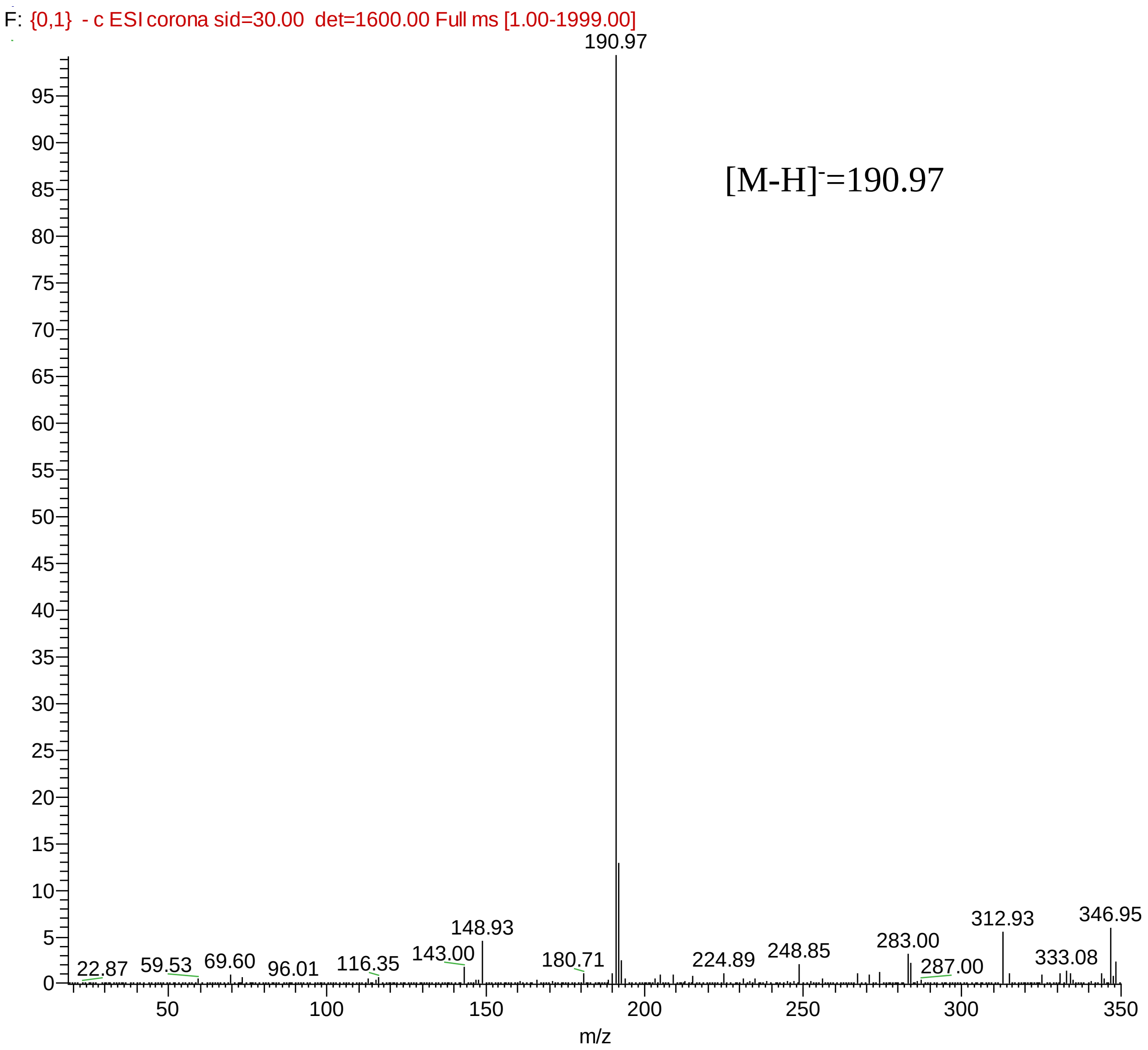

Figure S30. LRMS data of saccharonol A (**7**).

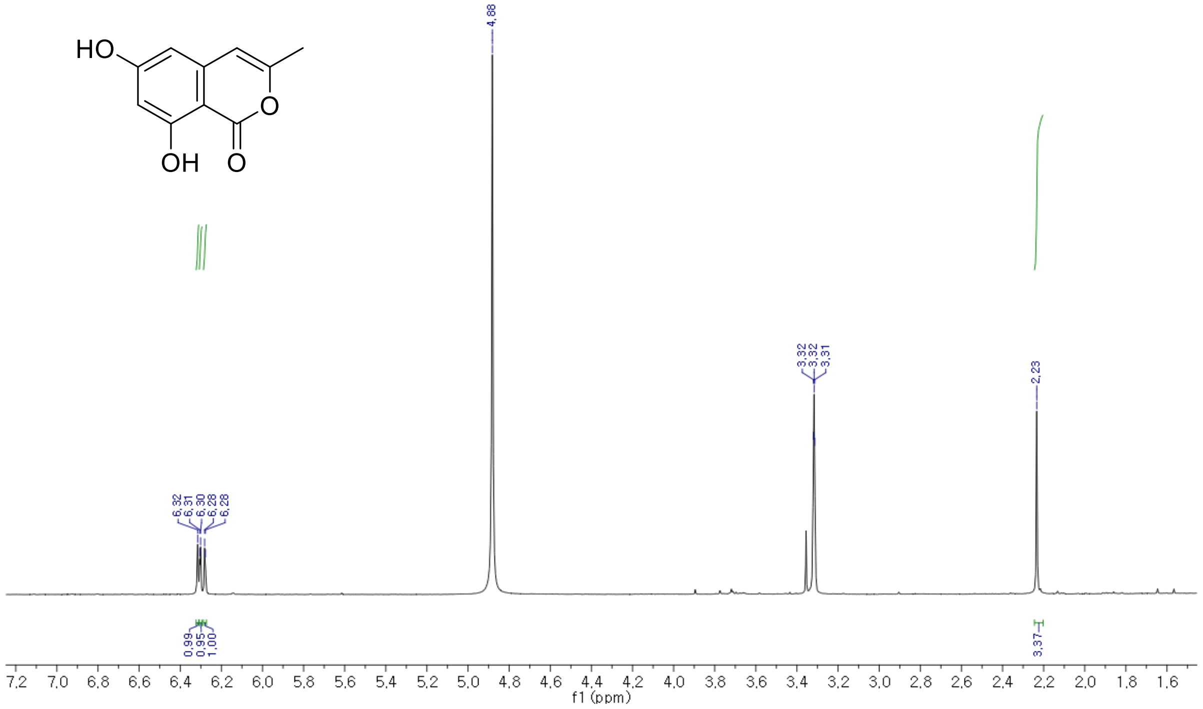

Figure S31. ^{1}H NMR spectrum of saccharonol A (**7**).

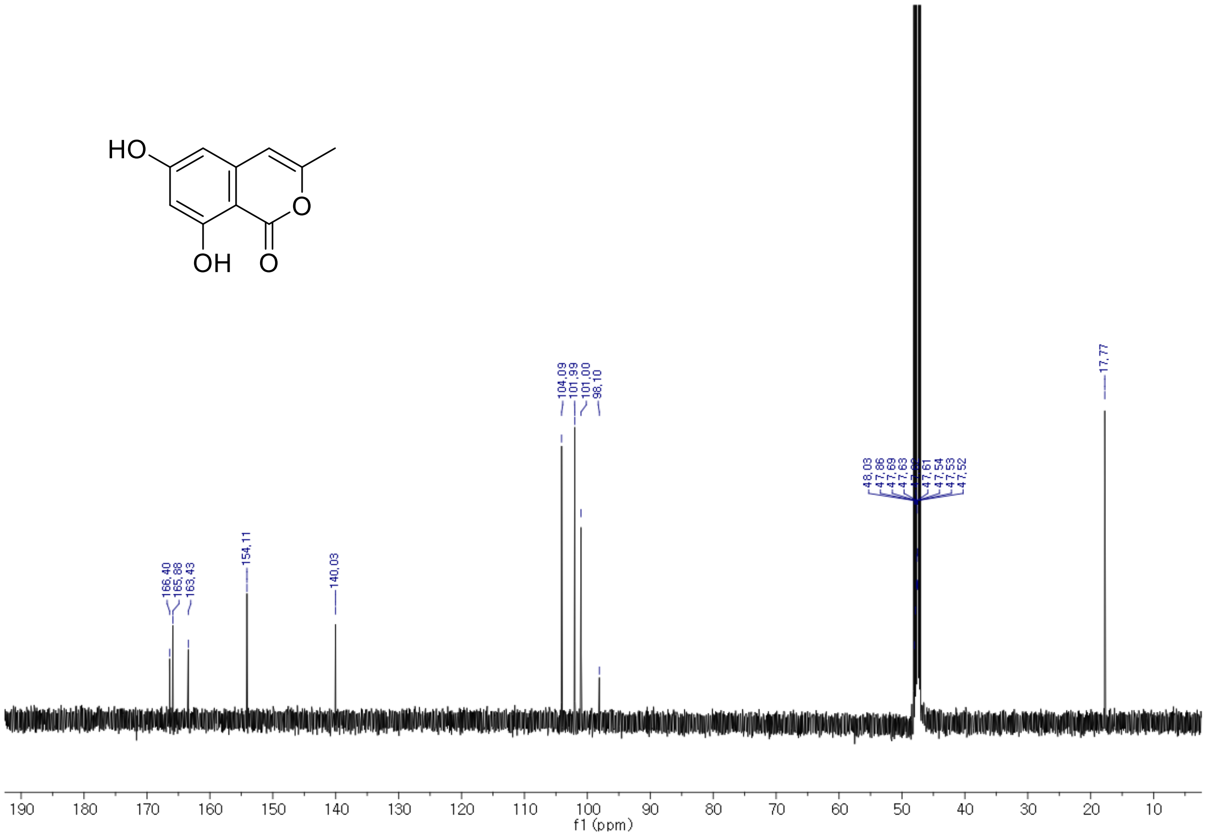

Figure S32. ^{13}C NMR spectrum of saccharonol A (**7**).

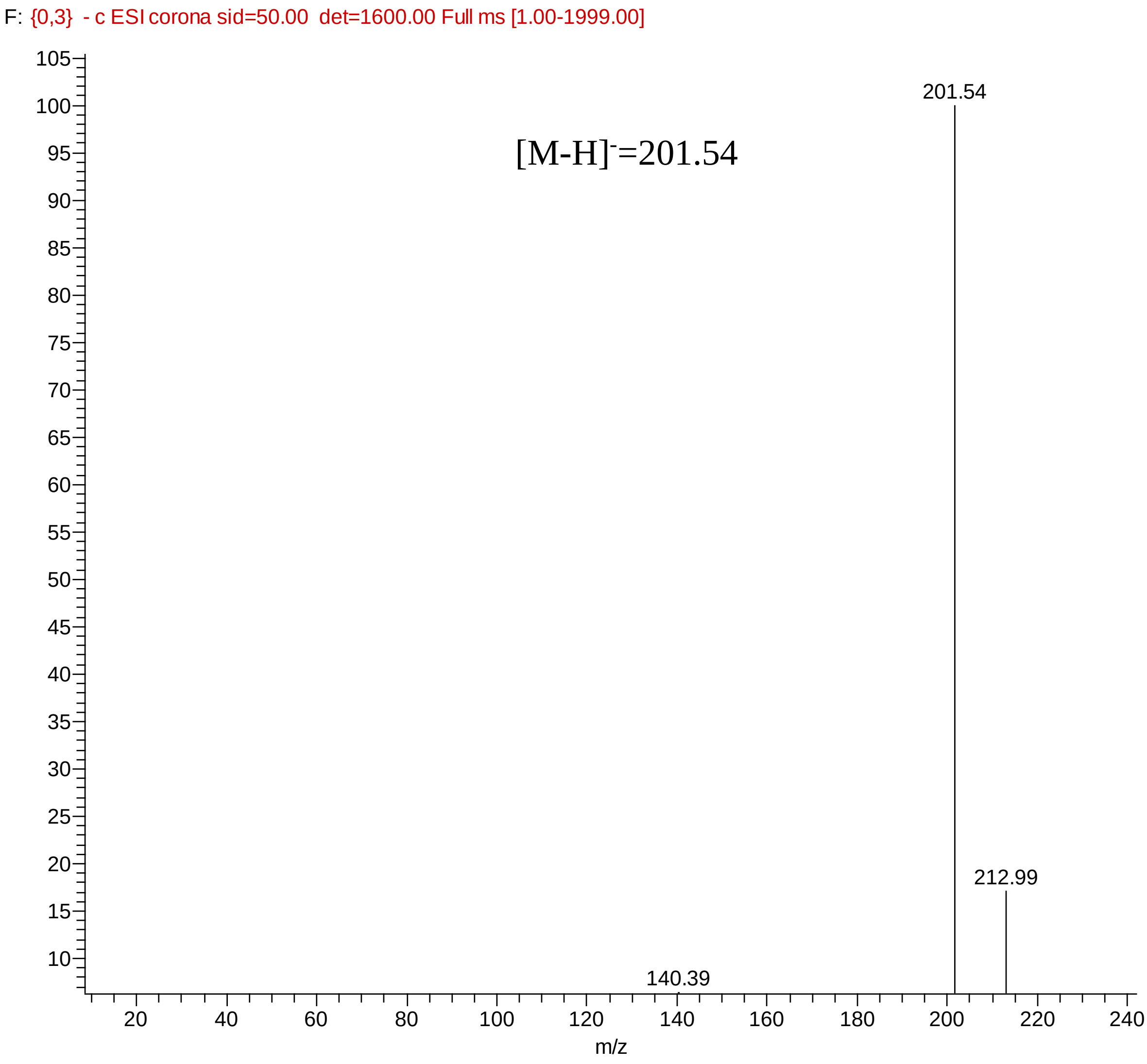

Figure S33. LRMS data of phomaligol D (**8**).

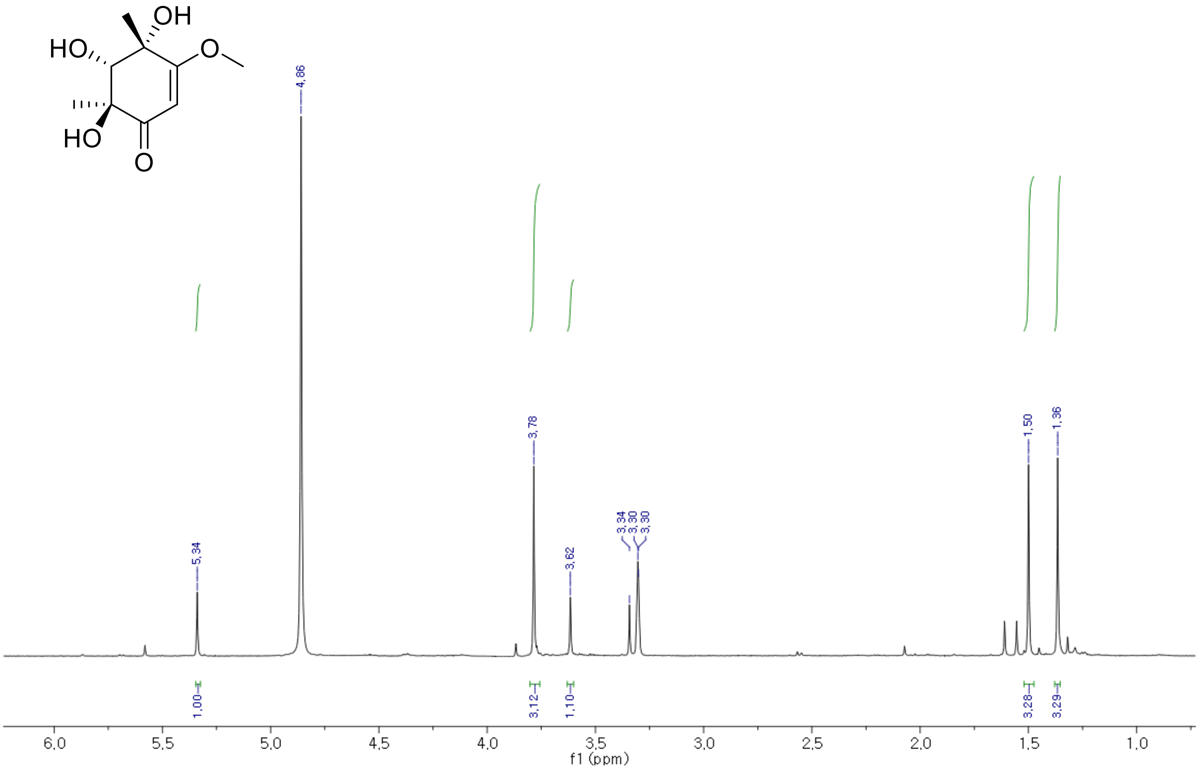

Figure S34. ^{1}H NMR spectrum of phomaligol D (**8**).

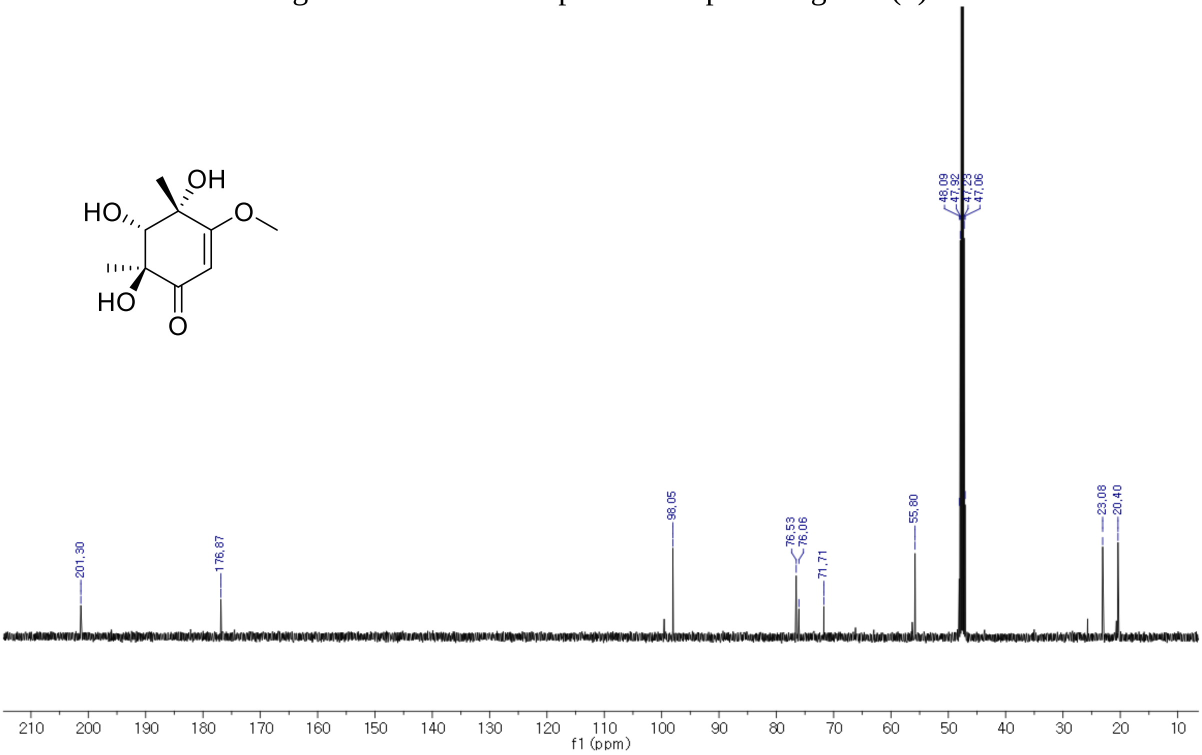

Figure S35. ^{13}C NMR spectrum of phomaligol D (**8**).

Figure S36. DFT optimized conformers and populations of **1** (2*R*, 5*R*, 7*S*) above 5% population.

Conformer 1

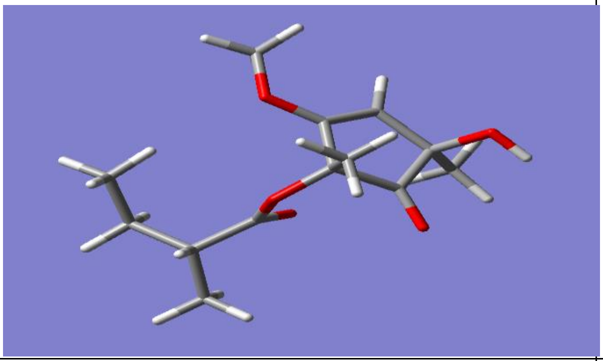

Conformer 2

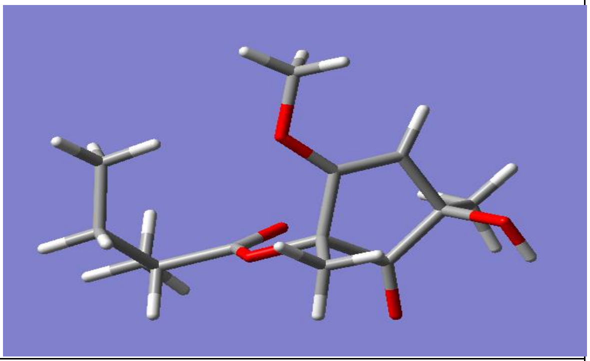

Conformer 3

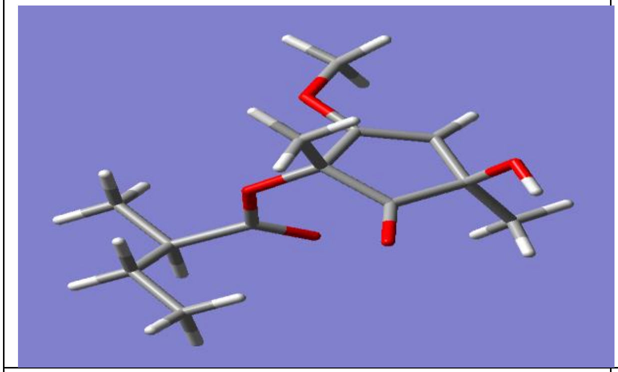

Conformer 4

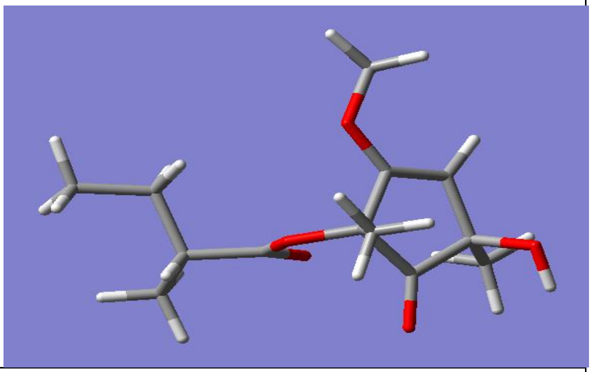

Conformer 5

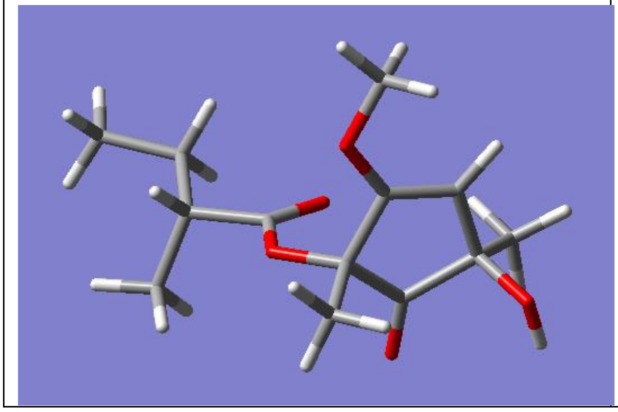

Table S1. Gibbs free energies and Boltzmann distribution of conformers of compound **1**.

B3LYP/6-31+G(d,p) Gibbs free energy (298.15K)		
	G (Hartree)	Population (%)
Conformer 1	-883.421988	41.48
Conformer 2	-883.422344	17.88
Conformer 3	-883.423125	16.88
Conformer 4	-883.422103	7.65
Conformer 5	-883.421843	5.99

Table S2. ECD calculation and energy minimized coordinates of conformer 1 for all atoms (Å).

Atom	X	Y	Z	Atom	X	Y	Z
C	1.9936	0.8811	-0.7801	H	1.7164	3.7205	-0.5405
C	0.9888	1.2109	0.0488	H	0.5433	3.1248	-1.7517
C	0.6175	0.1112	1.0196				
C	1.4338	-1.0738	0.4532				
C	2.4418	-0.5418	-0.5915				
O	-0.8083	-0.1603	1.0797				
C	1.0167	0.4156	2.4643				
C	-1.4069	-0.6886	-0.0112				
O	-0.7999	-0.9808	-1.0251				
C	-2.9044	-0.8567	0.1805				
C	-3.3230	-2.2717	-0.2486				
C	-3.6565	0.2268	-0.6295				
C	-3.4239	1.6594	-0.1401				
O	1.3736	-2.2146	0.8565				
C	2.4865	-1.3825	-1.8651				
O	3.7327	-0.5310	0.0592				
O	0.3034	2.3624	0.1772				
C	0.6747	3.4274	-0.7077				
H	2.4546	1.5329	-1.5113				
H	0.7832	-0.4455	3.0954				
H	2.0888	0.6172	2.5188				
H	0.4705	1.2897	2.8265				
H	-3.1225	-0.7143	1.2440				
H	-4.4001	-2.4002	-0.1096				
H	-3.0869	-2.4396	-1.3031				
H	-2.8102	-3.0375	0.3412				
H	-3.3674	0.1344	-1.6837				
H	-4.7256	-0.0096	-0.5772				
H	-3.9927	2.3705	-0.7472				
H	-3.7469	1.7762	0.9003				
H	-2.3686	1.9452	-0.1923				
H	2.7328	-2.4221	-1.6237				
H	1.5177	-1.3678	-2.3689				
H	3.2512	-0.9879	-2.5402				
H	4.0047	-1.4560	0.1636				
H	0.0123	4.2602	-0.4755				

Table S3. ECD calculation and energy minimized coordinates of conformer 2 for all atoms (Å).

Atom	X	Y	Z	Atom	X	Y	Z
C	1.9553	0.9146	-0.8104	H	1.4797	3.7364	-0.6894
C	0.9220	1.2092	-0.0034	H	0.3680	3.0111	-1.8880
C	0.6192	0.1291	1.0128				
C	1.5135	-1.0221	0.4972				
C	2.4958	-0.4659	-0.5593				
O	-0.7837	-0.2303	1.0848				
C	0.9953	0.5198	2.4432				
C	-1.3508	-0.8265	0.0097				
O	-0.7222	-1.1181	-0.9899				
C	-2.8329	-1.0825	0.2347				
C	-3.4338	-1.8659	-0.9365				
C	-3.5912	0.2321	0.5562				
C	-3.5079	1.3116	-0.5284				
O	1.5205	-2.1496	0.9408				
C	2.6108	-1.3514	-1.7975				
O	3.7754	-0.3436	0.1026				
O	0.1546	2.3127	0.0682				
C	0.4635	3.3643	-0.8561				
H	2.3769	1.5643	-1.5668				
H	0.8155	-0.3268	3.1105				
H	2.0523	0.7915	2.4888				
H	0.3934	1.3718	2.7679				
H	-2.8862	-1.7038	1.1389				
H	-4.4902	-2.0718	-0.7413				
H	-3.3593	-1.3101	-1.8747				
H	-2.9193	-2.8200	-1.0763				
H	-4.6394	-0.0341	0.7352				
H	-3.2055	0.6346	1.4980				
H	-4.0966	2.1872	-0.2375				
H	-2.4758	1.6459	-0.6785				
H	-3.8951	0.9600	-1.4897				
H	2.9232	-2.3619	-1.5130				
H	1.6488	-1.4216	-2.3098				
H	3.3546	-0.9332	-2.4817				
H	4.1046	-1.2439	0.2504				
H	-0.2586	4.1573	-0.6663				

Table S4. ECD calculation and energy minimized coordinates of conformer 3 for all atoms (Å).

Atom	X	Y	Z	Atom	X	Y	Z
C	2.3587	0.5298	-0.4847	H	2.8725	3.2984	0.0667
C	1.3370	1.1039	0.1732	H	1.8734	3.1747	-1.4110
C	0.4492	0.1140	0.8950				
C	0.9755	-1.2250	0.3299				
C	2.3099	-0.9716	-0.4086				
O	-0.9648	0.3157	0.6483				
C	0.5933	0.1655	2.4169				
C	-1.4410	0.0811	-0.5963				
O	-0.7401	-0.3123	-1.5095				
C	-2.9308	0.3614	-0.6905				
C	-3.2112	1.8645	-0.5064				
C	-3.7310	-0.4965	0.3136				
C	-3.4979	-2.0042	0.1740				
O	0.4801	-2.3118	0.5371				
C	2.3982	-1.6929	-1.7510				
O	3.3576	-1.4190	0.4822				
O	1.0058	2.3988	0.3218				
C	1.8535	3.3522	-0.3307				
H	3.1432	1.0492	-1.0200				
H	-0.0223	-0.6169	2.8678				
H	1.6367	0.0040	2.6969				
H	0.2707	1.1406	2.7894				
H	-3.2076	0.0675	-1.7086				
H	-4.2786	2.0604	-0.6446				
H	-2.9286	2.1945	0.4971				
H	-2.6595	2.4672	-1.2342				
H	-3.4829	-0.1734	1.3311				
H	-4.7931	-0.2696	0.1632				
H	-4.1281	-2.5597	0.8752				
H	-3.7369	-2.3519	-0.8372				
H	-2.4563	-2.2710	0.3835				
H	2.2615	-2.7706	-1.6112				
H	1.6224	-1.3346	-2.4310				
H	3.3804	-1.5182	-2.1993				
H	3.3172	-2.3878	0.5028				
H	1.4257	4.3323	-0.1228				

Table S5. ECD calculation and energy minimized coordinates of conformer 4 for all atoms (Å).

Atom	X	Y	Z	Atom	X	Y	Z
C	2.0723	0.8820	-0.8286	H	1.6672	3.7210	-0.8067
C	1.0487	1.2314	-0.0310	H	0.5271	2.9822	-1.9692
C	0.7047	0.1873	1.0081				
C	1.5712	-1.0030	0.5348				
C	2.5699	-0.5070	-0.5369				
O	-0.7123	-0.1199	1.0608				
C	1.0690	0.6009	2.4344				
C	-1.2784	-0.7273	-0.0079				
O	-0.6370	-1.1177	-0.9651				
C	-2.7869	-0.8079	0.1405				
C	-3.3459	-1.9835	-0.6675				
C	-3.3874	0.5638	-0.2721				
C	-4.8777	0.7041	0.0504				
O	1.5558	-2.1124	1.0217				
C	2.6621	-1.4304	-1.7490				
O	3.8520	-0.4038	0.1243				
O	0.3140	2.3578	0.0113				
C	0.6405	3.3683	-0.9508				
H	2.5147	1.4967	-1.6022				
H	0.8545	-0.2242	3.1182				
H	2.1327	0.8429	2.4909				
H	0.4875	1.4774	2.7293				
H	-2.9939	-0.9554	1.2071				
H	-4.4267	-2.0653	-0.5314				
H	-3.1398	-1.8530	-1.7336				
H	-2.8957	-2.9286	-0.3504				
H	-2.8306	1.3557	0.2410				
H	-3.2213	0.7092	-1.3471				
H	-5.2329	1.7037	-0.2186				
H	-5.4871	-0.0200	-0.4983				
H	-5.0647	0.5594	1.1202				
H	2.9403	-2.4419	-1.4343				
H	1.7011	-1.4842	-2.2648				
H	3.4229	-1.0559	-2.4398				
H	4.1516	-1.3090	0.3022				
H	-0.0595	4.1859	-0.7830				

Table S6. ECD calculation and energy minimized coordinates of conformer 5 for all atoms (Å).

Atom	X	Y	Z	Atom	X	Y	Z
C	2.2784	0.6849	-0.7813	H	2.6369	3.4954	-0.3375
C	1.3891	1.1648	0.1048	H	1.3324	3.2602	-1.5376
C	0.7941	0.1023	1.0028				
C	1.3095	-1.1865	0.3219				
C	2.3956	-0.8128	-0.7132				
O	-0.6527	0.1643	1.0889				
C	1.2762	0.1902	2.4517				
C	-1.3781	-0.1374	-0.0133				
O	-0.8727	-0.4855	-1.0635				
C	-2.8682	-0.0161	0.2596				
C	-3.3108	-1.2023	1.1446				
C	-3.6534	0.0890	-1.0595				
C	-5.1389	0.4095	-0.8685				
O	0.9976	-2.3133	0.6401				
C	2.2300	-1.5383	-2.0461				
O	3.6652	-1.1448	-0.1055				
O	0.9789	2.4236	0.3419				
C	1.5524	3.4467	-0.4812				
H	2.8609	1.2722	-1.4797				
H	0.8624	-0.6437	3.0240				
H	2.3668	0.1382	2.4843				
H	0.9506	1.1332	2.8971				
H	-3.0052	0.9061	0.8384				
H	-4.3622	-1.0989	1.4225				
H	-3.1904	-2.1504	0.6098				
H	-2.7210	-1.2472	2.0633				
H	-3.1936	0.8680	-1.6782				
H	-3.5390	-0.8483	-1.6164				
H	-5.6307	0.5342	-1.8381				
H	-5.6673	-0.3856	-0.3337				
H	-5.2752	1.3394	-0.3047				
H	2.2347	-2.6225	-1.8903				
H	1.2840	-1.2642	-2.5178				
H	3.0558	-1.2757	-2.7135				
H	3.7209	-2.1126	-0.0769				
H	1.0941	4.3833	-0.1660				
